# An overview of current drugs and prophylactic vaccines for coronavirus disease 2019 (COVID-19)

**DOI:** 10.1186/s11658-022-00339-3

**Published:** 2022-05-13

**Authors:** Armina Alagheband Bahrami, Ali Azargoonjahromi, Samin Sadraei, Aryan Aarabi, Zahra Payandeh, Masoumeh Rajabibazl

**Affiliations:** 1 Department of Biotechnology, School of Advanced Technologies in Medicine, Shahid Beheshti University of Medical Sciences, Tehran, Iran; 2 Shiraz University of Medical Sciences, Shiraz, Iran; 3 Shahid Beheshti University of Medical Sciences, Tehran, Iran; 4 Department Medical Biochemistry and Biophysics, Division Medical Inflammation Research, Karolinska Institute, Stockholm, Sweden; 5 Department of Clinical Biochemistry, Faculty of Medicine, Shahid Beheshti University of Medical Sciences, Tehran, Iran

**Keywords:** SARS-CoV-2, COVID-19 pandemic, Prophylactic vaccines, Platform, Vaccination

## Abstract

Designing and producing an effective vaccine is the best possible way to reduce the burden and spread of a disease. During the coronavirus disease 2019 (COVID-19) pandemic, many large pharmaceutical and biotechnology companies invested a great deal of time and money in trying to control and combat the disease. In this regard, due to the urgent need, many vaccines are now available earlier than scheduled. Based on their manufacturing technology, the vaccines available for COVID-19 (severe acute respiratory syndrome coronavirus 2 (SAR-CoV2)) infection can be classified into four platforms: RNA vaccines, adenovirus vector vaccines, subunit (protein-based) vaccines, and inactivated virus vaccines. Moreover, various drugs have been deemed to negatively affect the progression of the infection via various actions. However, adaptive variants of the SARS-CoV-2 genome can alter the pathogenic potential of the virus and increase the difficulty of both drug and vaccine development. In this review, along with drugs used in COVID-19 treatment, currently authorized COVID-19 vaccines as well as variants of the virus are described and evaluated, considering all platforms.

**Graphical abstract:**

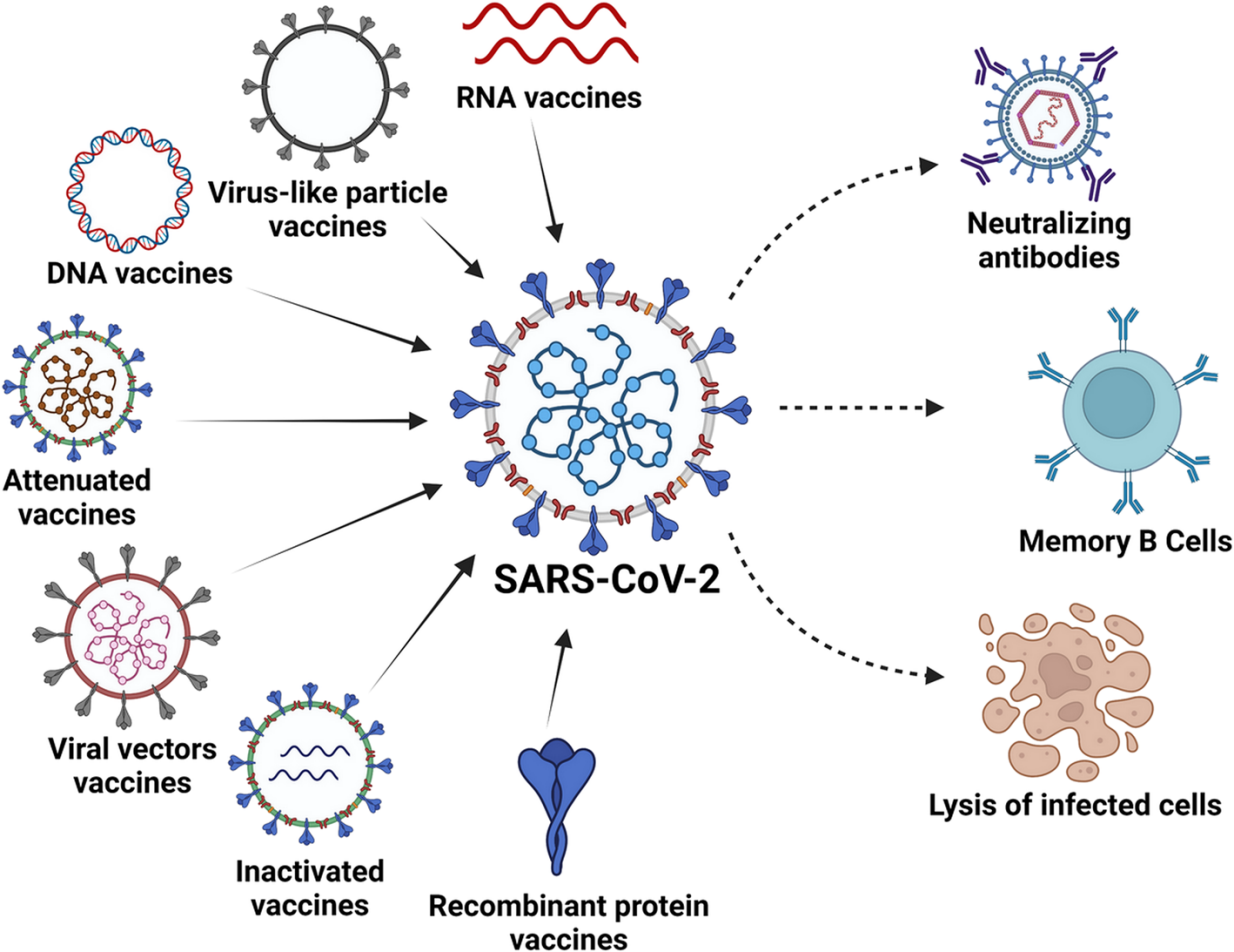

## Background

COVID-19, a contagious viral infection, can be passed on to others via inhalation of viral droplets as a result of coughing or sneezing. To protect persons against COVID-19, it is imperative to increase knowledge about the different characteristics of the virus causing COVID-19, such as its structure, function, and target human organs.

In terms of their structure, coronaviruses are crown-shaped with spikes, while their size is highly variable with average diameters of 80–160 nm. It has been found that the novel coronavirus-2019 (nCoV-2019) has a single-strand RNA genome with a length of 29.9 kb [1]. The nCoV-2019 genome encodes four structural proteins: spike (S), envelope (E), membrane (M), and nucleocapsid (N), as well as six accessory proteins (3a, 6, 7a, 7b, 8, and 9b) [2].

Regarding its function and the organs targeted, upon entering the host, the nCoV-2019 virus can infect macrophages by attaching S proteins to dipeptidyl peptidase-4 (DPP4) on the host cell, thereby releasing genomic RNA into the cytoplasm. DPP-4 can suppress NLRP3, TLR4, and interleukin (IL)-1β in human macrophages through inhibition of protein kinase C (PKC) activity. This can lead to the presentation of CoV antigens to T cells by macrophages. Such antigen presentation not only culminates in the provocation of both activation and differentiation of T-cells but also leads to the generation and consequent release of large amounts of cytokines to reinforce the immune response.

Of note, although coronaviruses have the capability to induce ongoing production of mediators that negatively affect the activation of both NK and CD8 T-cells, a large amount of cytokines produced by CD8 T-cells can obliterate viruses [3]. TLR‐3 sensitized by dsRNA, interferon regulatory factors (IRFs), and NF‐κB signaling pathways can be activated in such a condition, hence producing both type I IFNs and proinflammatory cytokines. It is worth noting that the secretion of antiviral proteins protecting uninfected cells can be elevated by the elevated amount of type I IFNs.

During the replication of coronaviruses, virus accessory proteins disturb TLR‐3 signaling by attaching to the dsRNA to hinder TLR‐3 activation, thereby escaping from the immune system. There is a likelihood that TLR‐4 is capable of identifying S protein, which per se results in activation of proinflammatory cytokines through the MyD88-dependent pathway. Indeed, an immune response to dsRNA will be enhanced when the viral genome commences to replicate.

During a coronavirus infection, immune mediators are induced as a result of the virus–host interaction, promoting the secretion of both chemokines and cytokines in infected cells. This event, in turn, summons both leukocytes and lymphocytes to the viral infection site for virus clearance. Therefore, antiviral drugs having low therapeutic efficacy and drug resistance can be supplanted by immunotherapy [2].

Recent studies carried out on the physicochemical properties of nCoV-2019 have highlighted that nCoV-2019 can be inactivated upon exposure to 56 °C for 30 min, ultraviolet radiation, using lipid solvents such as 75% ethanol, chlorine-containing disinfectant, peroxyacetic acid, and chloroform [4]. In addition, there are various types of vaccine that act in distinct ways to offer protection. All these types of vaccines, despite their distinct mechanisms, supply “memory” T-lymphocytes as well as B-lymphocytes, thereby remembering how to fight the virus in the future. A few weeks is required after vaccination to produce both T-lymphocytes and B-lymphocytes. This is why it is possible for a person to be infected with COVID-19 on exposure to the virus until suffiient time has passed to provide protection by the vaccine, before or just after vaccination.

On the whole, the general pathophysiology of SARS-CoV-2 is as follows: SARS-CoV-2 can be passed on to others through coughing and sneezing. The virus then enters the lungs via the respiratory tract and subsequently attacks alveolar epithelial type 2 (AT2) cells, in addition to the AT2 cells that are responsible for producing surfactant to decrease the surface tension in alveoli, thus reducing the collapsing pressure. It has been reported that the spike proteins of SARS-CoV-2 bind to the ACE-2 receptors on AT2 cells [5, 6]. Upon entering a host cell, the virus releases its positive-sense ssRNA using the host cell ribosome to produce polyproteins. The ssRNA can also use RNA-dependent RNA polymerases to duplicate its RNA. Synthesized spike proteins can be distributed to vesicle carriers via the cell packaging structure. The proteinases in the cytoplasm cleave the synthesized polyproteins of SARS-CoV-2 [7].

**Fig. 1:**
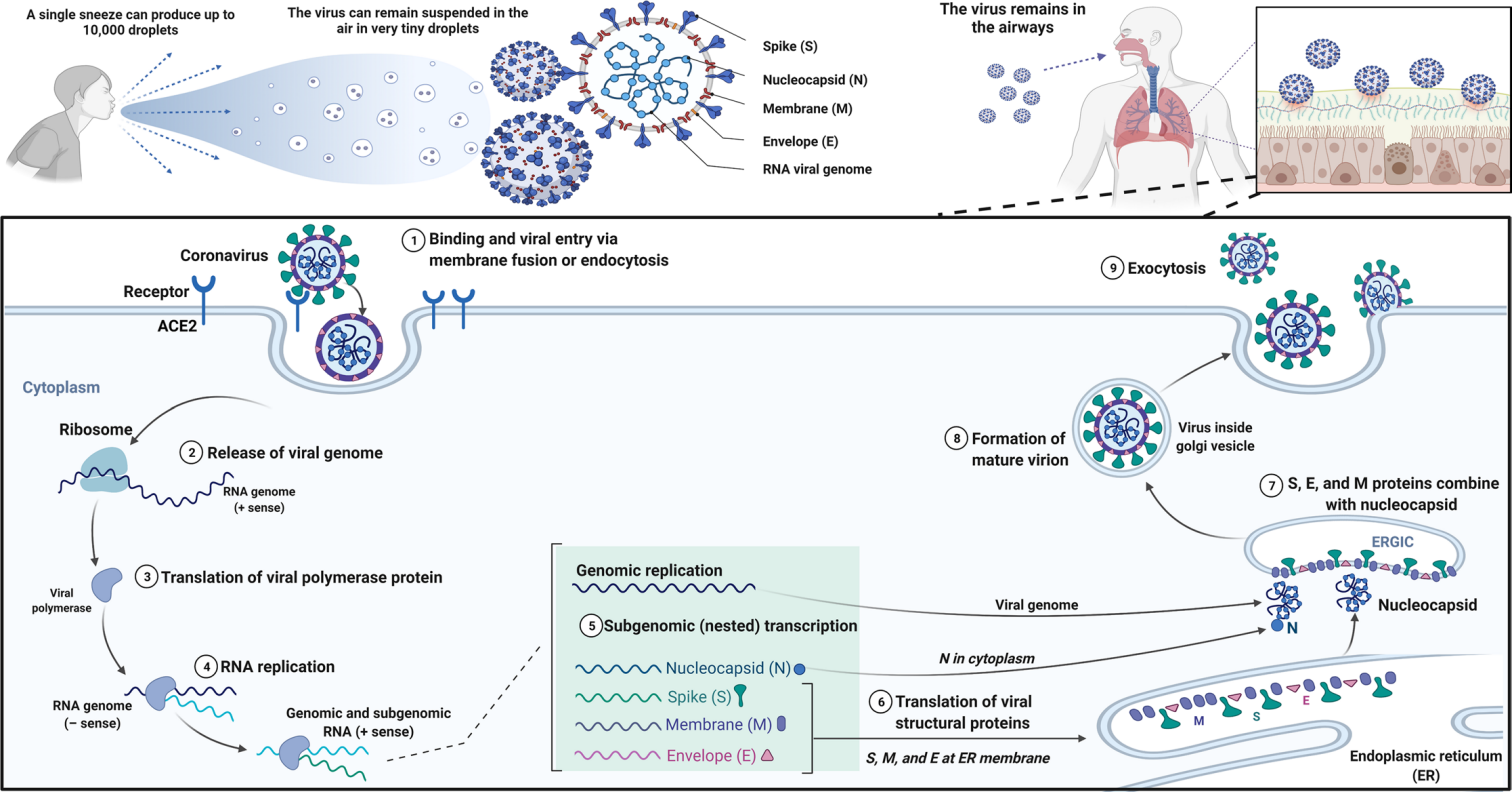
The coronavirus replication cycle. Binding of the virion to the host cell receptor through its spike protein S1 subunit is the first step of the coronavirus life cycle. After receptor binding, the virus gains access to the cytosol by acid-dependent proteolytic cleavage of the S protein into S1 and S2 subunits. Then, after release of the viral genome, the replicase can be translated from the genomic RNA. Following this, viral RNA synthesis and viral replication–transcription complexes occur. Over the next step, viral structural proteins (S, E, and M) are translated from the RNA, thus inserting into the endoplasmic reticulum and moving to the endoplasmic reticulum–Golgi intermediate compartment (ERGIC). Then, multiple copies of the nucleocapsid (N protein) package genomic RNA can be seen. They interact with hydrophobic M proteins in the ERGIC, thereby serving direct assembly of the virion. Virions budded from the membranes of the ERGIC are then transported out of the cell through the exocytic pathway

Further, the virus also releases specific inflammatory mediators to provoke macrophages, thereby releasing cytokines (IL-1, IL-6, and TNFα) [8] and chemokines (CCL2 and CXCL10) into the bloodstream. The implication of releasing such molecules is to increase both vasodilation and capillary permeability; therefore, plasma will leak into the interstitial spaces of the alveoli cells, resulting in a noticeable decline in surfactant levels in AT2 and compressing alveoli cells [9–13]. The cascade events eventually culminate in alveolar collapse and impaired gaseous exchange. Simultaneously, inflammatory cytokine (cytokine storm) secretion can be shown [14]; the production and recruitment of neutrophils and macrophages which use IL-21, IL-22, and IL-17 are escalated by inflammatory mediators [9]. In the later stages of the disease, all these steps lead to difficulty in hypoxemia, breathing, and cough. A schematic representation of the coronavirus replication cycle is shown in Fig. 1.

Although effective vaccines and drugs have provided an opportunity to protect against or treat COVID-19, as illustrated in this review, viral variants can pose great challenges to these current vaccines and drugs. Therefore, the importance of vaccination will escalate if SARS-CoV-2 variants show increased transmissibility or virulence. Some convergent changes of the key amino acids in the S protein have occurred. That is why, to cover multiple variants, vaccines should contain such selected residues. To date, there is insufficient evidence to show that these variants have entirely escaped from the vaccines [15]. SARS-CoV-2 variants, categorized into two types, are described below.

## Main text

### Current drug treatments

A patient afflicted with COVID-19 ought to, first of all, have both bed rest and water intake to save calories and diminish the risk of dehydration, respectively. In the situation where the patient hjas a high fever (> 38.5 ℃), antipyretic drugs such as ibuprofen or acetaminophen, as needed, are recommended.

Various antiviral drugs have been introduced by the National Health Commission of China (NHC) for the prevention, diagnosis, and treatment of COVID-19. The epitomes of such drugs are interferon α (IFN-α), lopinavir/ritonavir, chloroquine phosphate, ribavirin, and arbidol, as presented in the following paragraphs (Table 1).

**Table 1:** Drugs used to treat COVID-19 and corresponding information

Drug	Function	Dosage	Administration method	Refs.
Interferon α (IFN-α)	Hindering virus replication	5 million U, 2 times/day for adults	Inhalation	[17, 51]
Lopinavir/ritonavir	Inhibiting cytochrome P450 functions	200 mg/50 mg/capsule, 2 capsules each time, 2 times/day	Orally	[16, 17]
Chloroquine phosphate	Binding to the nuclear protein, thus preventing transcription of RNA	500 mg (300 mg for chloroquine) for adults, 2 times/day	Orally	[1, 52]
Hydroxychloroquine	Increasing endosomal pH, preventing virus–cell fusion, and interfering with glycosylation of the ACE2 receptor	800 mg once daily on day 1, followed by 200 mg twice daily for 7 days	Orally	[27, 53]
Dexamethasone	Inhibiting a proinflammatory gene being responsible for encoding chemokines, cytokines, and cell adhesion molecules (CAM)	6 mg once daily for up to 10 days	Orally, intravenous	[34, 54]
Budesonide	Decreasing the level of both IL-6 and antiphospholipid antibodies	800 mcg BID (FDA approved max. 720 mcg BID)	Inhalation	[42, 55]
Ribavirin (tribavirin)	Inhibiting viral replication	500 mg for adults, 2 to 3 times/day	Intravenous infusion	[16, 56]
Arbidol	Hampering entry of viral genes into the nucleus and blocking trimerization of S protein	200 mg for adults, 3 times/day	Orally	[57, 58]
Remdesivir	Binding to RdRp and acting as an RNA chain terminator	200 mg, loading dose on day 1, followed by 100 mg IV daily for up to 10 days, infused over 30–60 min	Intravenous infusion	[29]
Ciclesonide	Blocking viral replication	320 µg twice daily	Inhalation	[59]
Favipiravir	Acting as a RdRp inhibitor, and hindering both the replication and transcription of the viral RNA chain	1800 mg twice a day on day 1, followed by 800 mg twice a day maximum up to 14 days	Orally	[60]
Paxlovid	Inhibiting viral replication	300 mg nirmatrelvir (two 150 mg tablets twice daily for 5 days)	Orally	[45, 46]
Molnupiravir (Lagevrio/Molulife)	Inhibiting replication of RNA viruses	Four 200 mg capsules every 12 h for 5 days, for a total of 40 capsules	Orally	[47, 48]
Monoclonal antibodies (bebtelovimab)	Preventing target cell binding and/or fusion and facilitating target cell death	175 mg given as an intravenous injection over at least 30 s	Intravenous	[50, 61]

#### Clinical trial drugs

It has been shown that IFN can disturb virus replication and increase the antiviral ability of cells. IFN-α, deemed to be a type I IFN, is capable of inhibiting RNA virus replication, providing a golden opportunity for its use via inhalation as a trial treatment against COVID-19 in the Diagnosis and Treatment Protocol for Coronavirus Pneumonia (DTPNCP) [16]. The dosage of IFN-α is 5 million U for 2 times/day for adults, while the method of administration is vapor inhalation [17].

Lopinavir/ritonavir (with ritonavir as a booster) has been known as a human immunodeficiency virus (HIV) protease inhibitor. It can inhibit cytochrome P450 functions, thus enhancing the antiretroviral activity against the virus. A study carried out on 47 patients suffering from COVID-19 showed that administration of combined lopinavir/ritonavir with pneumonia-associated adjuvant drugs resulted in hopeful outcomes, including reducing the body temperature and retrieving physiological mechanisms with neither evident toxic nor adverse events [18]. The dosage of lopinavir/ritonavir is 200 mg/50 mg/capsule, 2 capsules each time, 2 times/day, and the drug should be taken orally [17].

Chloroquine phosphate, which is taken to treat malaria and rheumatic diseases, has been shown to have broad-spectrum antiviral effects. It can bind to the nuclear protein and prevent transcription of RNA, as well as acting as an autophagy inhibitor. Accordingly, the rate of viral RNA clearance in the chloroquine group was significantly higher and faster than in the nonchloroquine group after 14 days, reaching 95.9% and 79.6%, respectively, but without any differences in the rate of adverse reactions between the two groups [16]. Chloroquine phosphate is taken orally at a dosage of 500 mg (300 mg for chloroquine) for adults, 2 times/day [17].

Furthermore, preliminary studies have indicated that hydroxychloroquine may be useful in the treatment of patients with COVID-19 [19, 20]. Hydroxychloroquine was found to have antivirus, antiinflammation, and antithrombotic functions. However, to date, no immunity-bolstering impact of this drug has been reported. Hydroxychloroquine, although reported to inhibit SARS-CoV-2 in vitro [21, 22], has not yet been shown to exhibit a convincing anti-SARS-CoV-2 effect in vivo [23, 24]. Of note, on 28 March 2020, the US Food and Drug Administration permitted an emergency use authorization of hydroxychloroquine in hospitalized patients with COVID-19 [25]. However, after a while, the US Food and Drug Administration (FDA) issued precautions regarding the use of this drug for treating COVID-19. This was because of reports of serious heart rhythm problems and other safety issues, such as blood and lymph system disorders, kidney injuries, and liver problems and failure [26]. Hydroxychloroquine has the ability to augment endosomal pH, prevent virus–cell fusion, and interfere with glycosylation of the ACE2 receptor, thereby inhibiting binding of the SARS-CoV-2 S protein to ACE2 [27]. Moreover, this drug can prevent antigen processing and suppress inflammatory signaling pathways that reduce the production of proinflammatory cytokines such as TNF-α, IL-6, and IFN-γ [28], and IFN-α and CCL4 [26].

Ribavirin, also known as tribavirin, is another drug being considered for use to treat patients afflicted with COVID-19. It is a purine nucleoside analog that is deemed a broad-spectrum nucleoside antiviral drug. It has direct and indirect functions giving rise to inhibition of viral replication. This drug targets the RNA-dependent RNA polymerase (RdRp) of SARS-CoV-2 [16]. Ribavirin should be taken through intravenous infusion at a dosage of 500 mg for adults, 2 to 3 times/day, and can also be administered in combination with either IFN-α or lopinavir/ritonavir [17].

Another drug that can be used in cases of COVID-19 is arbidol, a nonnucleoside antiviral drug. Arbidol shows a wide field of action, including the ability to hamper the entry of viral genes into the nucleus and to hinder the contact and fusion of the viral envelope with the host cell membrane. Furthermore, arbidol has been shown to have inhibitory activity against SARS-CoV-2. It has been suggested that it may target the S protein, thus blocking its trimerization [16]. Arbidol is administered orally at a dosage of 200 mg for adults, 3 times/day [17]. In comparison with lopinavir/ritonavir, no viral load was seen among cases taking arbidol, while the opposite was true for patients administrated lopinavir/ritonavir after 14 days, which accounted for 44.1% [16].

In addition to these findings, various other drugs such as remdesivir, ciclesonide, and favipiravir can be employed to treat patients suffering from COVID-19, though further investigations are required to prove their outcomes. Prominent features of these drugs are discussed in the following paragraphs.

Remdesivir (RDV) (GS-5734), which is a monophosphoramidate prodrug of an adenosine analog, can bind to RdRp and act as an RNA chain terminator [29]. Both the safety and pharmacokinetics of RDV have been assessed in single- and multiple-dose phase intravenous infusions at doses of 3 mg and 225 mg, confirming that it is well tolerated and does not show kidney or liver toxicity, thereby diminishing the rate of mortality among COVID-19 patients. Notably, RDV has been shown to have a linear pharmacokinetic function solely at this dosage, while its intracellular half-life is more than 35 h [29]. It has been shown that remdesivir may be more effective than lopinavir/ritonavir combined with interferon-β to treat patients suffering from COVID-19. It was also shown that it can remarkably decrease the virus titer in mice infected with Middle East Respiratory Syndrome (MERS)-CoV, and ameliorate lung tissue damage. These findings lead to the prevailing notion that remdesivir can be considered the best potential drug to treat patients afflicted with COVID-19 [30]. The recommended dosage under investigation for treatment of COVID-19 is 200 mg intravenously (IV) loading dose on day 1, followed by 100 mg IV daily for up to 10 days, infused over 30–60 min [29]. Note that remdesivir is not recommended for patients diagnosed with moderate to severe COVID-19 and not requiring respiratory support. In contrast, remdesivir can be useful to reduce the time of recovery processes and the risk of progression among those in the early stage of the illness (≤ 10 days) and diagnosed to have high risk of hyperinflammation and requirement for supplemental oxygen [31].

Favipiravir is another drug that can be administrated to treat patients with COVID-19. It is a purine nucleic acid analog, acting as a RdRp inhibitor [32]. Of note, phosphoribosylation of favipiravir can be triggered by the host cell enzyme, thus producing bioactive favipiravir furylribo-5-triphosphate-inositol (favipiravir RTP). Insertion of favipiravir RTP into the viral RNA chain can occur as a result of the viral RNA polymerase misrecognizing favipiravir RTP, and subsequently favipiravir RTP can bind to the viral RNA polymerase domain, thereby hindering both the replication and transcription of the viral RNA chain [16]. Incidentally, respiratory system recovery begins upon administration of favipiravir, while the therapeutic process takes up to 19 days, resulting in withdrawal of ventilator support [33].

Dexamethasone, moreover, is a corticosteroid that is used in a wide range of conditions for its antiinflammatory and immunosuppressant effects. Dexamethasone is capable of inhibiting a proinflammatory gene that is responsible for encoding chemokines, cytokines, and cell adhesion molecules (CAM) [34]. The main action of this drug is to cause both immunosuppression and antiinflammatory effect [34, 35]. In one study, inhaled corticosteroids were shown to impair viral replication of SARS-CoV-2 [36], and also they can downregulate expression of the receptor (ACE2) used by SARS-CoV-2 to enter target cells [37]. In short, this drug has been shown to have benefits for patients with COVID-19, although further studies are required [38].

Budesonide, known by its brand name Pulmicort, is a corticosteroid whose inhaled form is used in chronic obstructive pulmonary disease (COPD) and asthma [39]. Budesonide was found to be capable of stabilizing the endothelium and decreasing the level of both IL-6 and antiphospholipid antibodies, all of which play important roles in COVID-19 infection [40, 41].

Furthermore, in another study, inhaled budesonide was found to reduce the requirement for urgent medical care while enhancing the clinical recovery of patients afflicted with COVID-19 [42]. Despite these findings, it is believed that further studies are required to reach a consensus on whether this drug can be applied to improve recovery and reduce disease progression in COVID-19 infection [43].

In addition to those mentioned above, another drug that is deemed to have a noticeable antiviral impact in the treatment of patients afflicted with COVID-19 infection is a corticosteroid named ciclesonide, which is taken via inhalation. According to a study carried out on patients afflicted with pneumonia with lymphocyte counts at or below the cutoff value of 978.1 cells/mm^3^, it was shown that remarkably fewer patients required ventilator support or intubation when on ciclesonide (11.18% versus 83.33%). Moreover, the lymphocyte count was significantly higher in ciclesonide-treated cases in the nonsevere pneumonia group than in the pre-ciclesonide condition. Ciclesonide has thus been considered for use in the treatment of COVID-19 in such conditions [44].

#### Authorized drugs

In comparison with placebo, it was reported by Pfizer Inc. that oral antiviral drug paxlovid (PF-07321332; ritonavir) has been shown to be able to remarkably reduce hospital admissions (by 89%), among people with COVID-19, in particularly among those at high risk of severe illness. In a study, trial participants were randomized, with half receiving paxlovid and the other half receiving a placebo. After 3 days of symptom onset, two groups of participants were received paxlovid and placebo. Less than 1% of patients who recieved paxlovid were admitted to hospital—up to 28 days, and also no deaths was reported among them. PF-07321332 (paxlovid; ritonavir) has been designed to hinder the activity of the SARS-CoV-2 3CL protease, an enzyme that the coronavirus needs to replicate. Paxlovid (PF-07321332; ritonavir) can also inhibit viral replication at a stage known as proteolysis, which occurs before viral RNA replication. For the time being, Pfizer Inc. (www.pfizer.com/news/press-release/press-release-detail/pfizers-novel-covid-19-oral-antiviral-treatment-candidate) plans to submit these data as part of its ongoing rolling submission to the US FDA for emergency use authorization (EUA) as soon as possible [45, 46].

In addition to the aforementioned, molnupiravir, under the brand name Lagevrio/Molulife, is deemed to be an antiviral drug. It can inhibit replication of RNA viruses, which is why molnupiravir can be used in the treatment of patients suffering from COVID-19 infection. Molnupiravir exerts its antiviral function by interfering with viral RNA replication [47, 48]. The antiviral drug molnupiravir has been shown to diminish the risk of hospital admission and death by approximately 50% among nonhospitalized adults with mild to moderate COVID-19 infection and who were at risk of poor outcomes [49]. This drug is administered as four 200 mg capsules taken orally every 12 h for five days, for a total of 40 capsules.

According to www.fda.gov/news-events/press-announcements/coronavirus-covid-19-update-fda-authorizes-additional-oral-antiviral-treatment-covid-19-certain, molnupiravir is not authorized to be taken for longer than 5 consecutive days. Although this drug has many benefits for adult patients with COVID-19, based on findings from animal reproduction studies, molnupiravir may cause fetal harm when administered to pregnant individuals. Therefore, molnupiravir is not recommended for use during pregnancy.

Of interest, monoclonal antibodies, which can be produced by multiple methods, but in particular in the laboratory by cloning unique white blood cells, have been shown to have a noticeable impact in the treatment of COVID-19 patients. Monoclonal antibodies have recently been approved by the FDA via emergency use authorizations among nonhospitalized patients with mild to moderate COVID-19. Monoclonal antibodies show various actions to interfere with viral pathogenesis, from binding to the spike protein of the virus, which prevents target cell binding and/or fusion, to facilitating target cell death by either activation of membrane attack complex (MAC) or antibody-dependent cytotoxicity [50]. However, it is of note, in accordance with https://www.fda.gov/news-events/press-announcements/coronavirus-covid-19-update-fda-authorizes-new-monoclonal-antibody-treatment-covid-19-retains on 11 February 2022, that monoclonal antibodies such as bebtelovimab have not yet been authorized for use in patients who are hospitalized or require oxygen therapy because of COVID-19 infection. This authorization is because treatment with bebtelovimab has not been studied in patients hospitalized due to COVID-19.

### How vaccines work

It is noteworthy that an increasing amount of neutralizing antibodies (Nabs) directed at the S protein, mediating cellular binding, was seen in early studies of SARS-CoV-2 vaccine candidates in a rhesus macaque model, whereby eliciting NAbs responses was indispensable for vaccines with the highest efficacy. Nonetheless, non-NAbs have been reported to play a crucial role in the protection against SARS-CoV-2 through Fc-mediated effector functions, namely antibody-dependent phagocytosis, antibody-dependent cellular cytotoxicity, and antibody-dependent natural killer cell activation [62].

#### Antibody responses

To illustrate some general information about antibodies, it is of note that they are distinct for the S, M, E, N, and further viral proteins. Many of the antibodies are also strain or group specific, thus being able to recognize only some but not all SARS-CoV-2 viruses. The attachment of SARS-CoV-2 to host cells occurs by binding of the S protein to ACE2, which is the viral receptor on the host cells. Subsequently, the S protein is primed by host cell proteases, furin, and the serine proteases TMPRSS2 and TMPRSS4, whereby the viral and cellular membranes fuse, resulting in entry of the viral RNA into the host cell [63].

In addition, antibodies may be neutralizing, though most are not. Even though both IgM and IgG antibodies to SARS-CoV-2 can be detectable within 1–2 weeks after the onset of symptoms in most patients [64], numerous studies have pointed out that the amount of IgA responding to S protein soars far earlier [65]. The neutralizing antibody, being fiercely specific to both the S protein and 3a protein, of epitopes has been shown to be protected in several viral strains, suggesting the notion that vaccines eliciting such antibodies are protective against multiple strains [63]. The S protein, comprising S1 and S2 domains, is targeted by neutralizing antibodies in coronaviruses, not to mention that the magnitude of neutralizing antibody responses is positively related to disease severity.

Regarding the immunogenicity of the neutralizing antibodies aroused by vaccine candidates, three efficacy levels have been reported, with the inactivated and AdV5 vaccine candidates, ChAdOx1 nCoV-19 and mRNA candidates, and recombinant protein vaccine candidate classified as offering immunogenicity at the lower end, in the medium range, and at the high end, respectively. It is worth noticing that the inactivated and recombinant protein vaccines give the impression of being more tolerable, compared with mRNA vaccines, while the AdV-vectored vaccines have been placed at the end of this classification [66].

Incidentally, antibodies binding to the S1 receptor binding domain (RBD) can effectively block its binding to ACE2. Meanwhile, antibodies binding to the S1 and S2 domains prevent the change of the S protein conformation and its fusion to the cell membrane [65].

#### Cellular immunity

T cells, and memory cells thereof such as CD4^+^, can produce various molecules to respond to viral antigens, notable among which is the cytokine interferon γ [67]. CD4^+^ cells also have a pivotal role to play in optimal antibody responses and in CD8^+^ T-cell activation [64]. Accordingly, it has been pointed out that the amount of CD4^+^ cells having the ability to recognize only the S protein of COVID-19 is lower than those considered to be cross-reactive to other proteins of COVID-19 among unexposed individuals, being 35% and well over 40%, respectively [65]. Moreover, CD4^+^ cells promote both B-cell responses and antibody production, leading to potent immunity against SARS-CoV-2 [67].

**Fig. 2:**
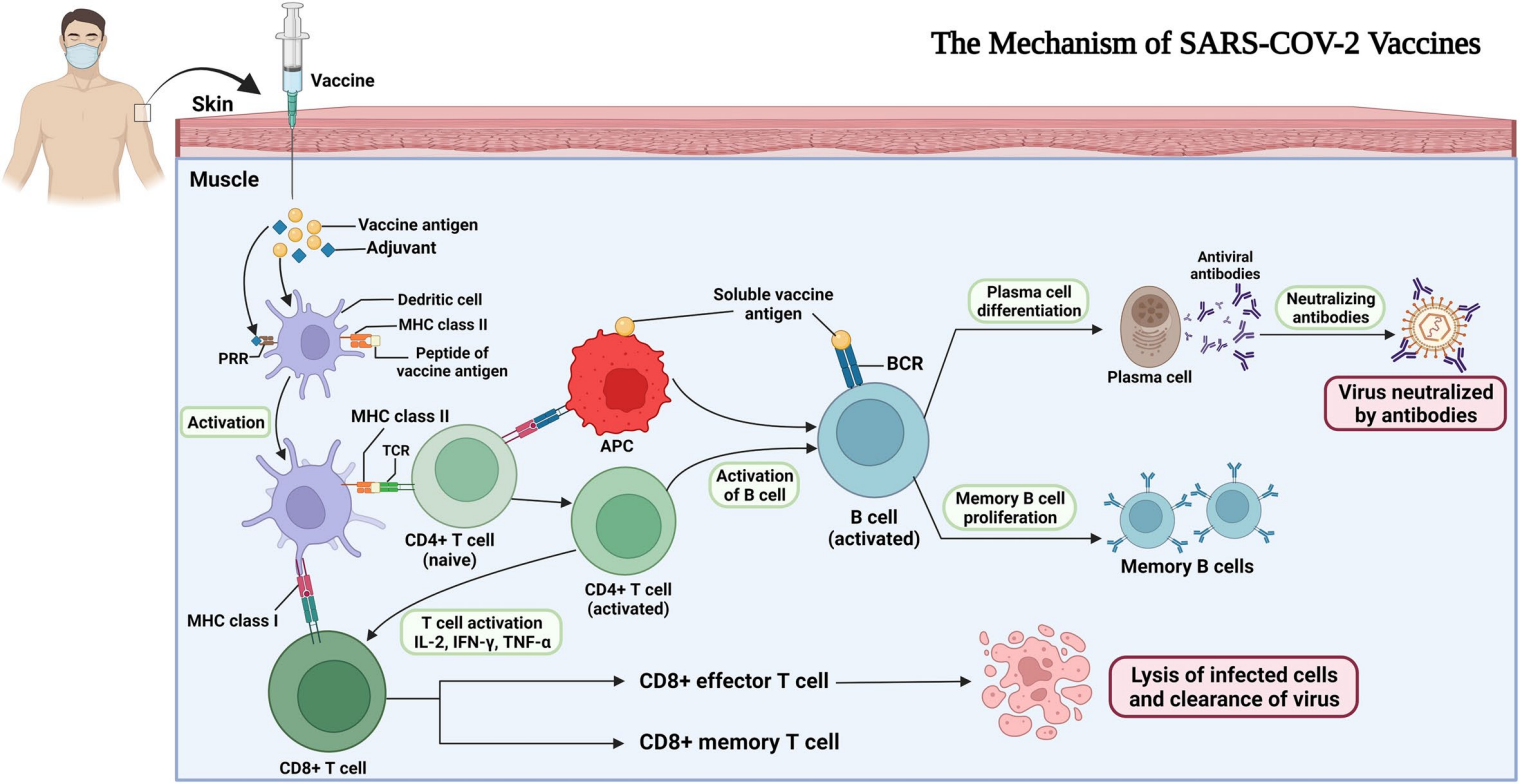
The immune system response to vaccination. The vaccine is injected intramuscularly, and subsequently the protein antigen is taken up by dendritic cells activated by pattern recognition receptors (PRRs) to danger signals in the adjuvant. Here, MHC molecules on the surface of dendritic cells can present peptides of the vaccine protein antigen, which in turn leads to activation of T cells via their T-cell receptor (TCR). Following this, B cells can be developed in the lymph node by T cells. This development is a result of the combination of soluble antigen with the B-cell receptor (BCR). The production of plasma cells produces a rapid rise in serum antibody levels over the next 2 weeks. Memory B cells are also generated, thus mediating immune memory. Of note, CD8^+^ memory T cells proliferate rapidly when they confront a pathogen, and CD8^+^ effector T cells also have a pivotal role to play in the obliteration of infected cells

The process of viral recognition by T cells, moreover, is mainly based on a wide variety of antigenic peptide/HLA complexes. This is because of the many differences between the human leukocyte antigen (HLA); that is, the HLA molecules that present T-cell antigens on the surface of APCs and those which do so on infected cells vary, not to mention that this variety is owing to the massive genetic HLA polymorphism, among most people [63]. The mechanism of SARS-CoV-2 vaccines and how the immune system is induced is illustrated in Fig. 2.

### Current vaccines

Based on the manufacturing technology applied, the vaccines available for COVID-19 (SAR-CoV-2) infection can be classified into four platforms: RNA vaccines, adenovirus vector vaccines, subunit (protein based) vaccines, and inactivated virus vaccines. The currently authorized COVID-19 vaccines are described below.

#### Pfizer–BioNTech

The Pfizer–BioNTech vaccine, also known as BNT162b2, is an mRNA vaccine encapsulated in a lipid nanoparticle formulation. The Pfizer vaccine can encode the prefusion S glycoprotein of SARS-CoV-2, the virus giving rise to COVID-19 [68].

According to the primary study, 170 confirmed cases of COVID-19 were evaluated, with 162 cases in the placebo versus 8 in the vaccine group. The efficacy of the Pfizer vaccine against COVID-19 began 28 days after the first dose was injected, reaching 95%. Of note, the efficacy of this vaccine has been found to be consistent in age, gender, race, and ethnicity categories, reaching over 94% among adults over the age of 65 years [69]. It is noteworthy that an interval between two doses of Pfizer vaccine of 21 days has been recommended, although intervals up to 42 days are deemed permissible [70].

The antibody neutralization level can be escalated by roughly 10, on average, via a booster (third) dose of the Pfizer vaccine, compared with the level after the second dose. The rate of confirmed infections showed a noticeable decline nearly 4–6 and 12 days after the booster dose, by a factor of 5.4 and 11.3, respectively. On the whole, this increased neutralization titer derived from the booster dose can both enhance the protection against infection and diminish the exacerbation of the disease [71].

However, the appropriate temperature to maintain this vaccine with high efficacy is −70 °C, and providing such an environment is a challenging issue [72].

Reactogenicity symptoms and adverse effects of the Pfizer vaccine among vaccine recipients, at the local injection site or as systemic reactions, have been reported frequently, mostly being mild to moderate, and lasting up to 7 days after vaccination. Systemic adverse reactions, among which the most common were fever, fatigue, headache, muscle pain, and chills, were reported 1–2 days, on average, after vaccination. These reactions were also seen more among recipients aged 18–55 years than those over 55 years [73].

Incidentally, such reactions have been more commonly reported after the second dose of the vaccine than the first [66]. Although it is still not clear whether the linkage between COVID-19 vaccination and thrombocytopenia is coincidental, some cases showing secondary immune thrombocytopenia (ITP) symptoms after Pfizer vaccination have been reported [74].

To date, the long-term immunity induced by the Pfizer vaccine remains unknown. There is also some evidence that the immunity derived from the Pfizer vaccine may dwindle, in particularly against new variants such as delta. This is why further studies are imperative to reach a consensus regarding the various characteristics of this vaccine [75, 76].

#### Moderna

The Moderna vaccine, which is known as mRNA-1273, is an mRNA-LNP (mRNA in liquid nanoparticles) vaccine, and it also includes three types of ingredients: (1) messenger ribonucleic acid (mRNA), (2) lipids (fats), and (3) salt, sugar, acid stabilizers, and acid. The Moderna vaccine is in many ways akin to the Pfizer–BioNTech vaccine, encoding the prefusion S protein. The final aim of this type of ingredient is to instruct the body to build a harmless piece of a protein from the virus giving rise to COVID-19. This protein can induce an immune response against COVID-19. As one of the ingredients of the Moderna vaccine, lipids can help the mRNA enter cells. The final type of ingredients are sugar, salt, acid stabilizers, tromethamine, and tromethamine hydrochloride that contribute to the stability of the vaccine molecules during the manufacturing, freezing, transport, and storage processes [77].

The Moderna vaccine is administered in two doses with an interval of 28 days [78]. A booster dose can also be administrated at least 6 months after taking the second dose [79]. Owing to the delicate genetic material in the vaccine, the Moderna vaccine ought to be kept in a very cold environment, with temperature between −25 and –15 °C. Upon defrosting, the vaccine should be kept at a temperature between 2 and 8 °C for 30 min to 2 h, and vaccine vials can also be stored for up to 30 days prior to being punctured; however, vials ought to be discarded if kept in the refrigerator for longer than 30 days [80, 81].

The efficacy reported in the study of the Moderna vaccine was remarkably acceptable, leading to its approval by the FDA in emergency conditions. To clarify this approval statistically, a study carried out in 30,420 participants with 185 participants in the placebo group and 11 in the mRNA-1237 group, the percentage protection in the age ranges of 18–65 and over 65 years was reported to be 95.6% and 86.4%, respectively [79].

The side effects of the Moderna vaccine (along with pain, redness, and swelling in the arm injected) have been shown to last up to 7 days after vaccination, namely tiredness, headache, muscle pain, chills, fever, and nausea. These side effects appear within 1–2 days after vaccination, being deemed normal signs of protection processes induced by the body [77, 82].

#### CureVac

CureVac, also known as CVnCoV, is another mRNA-based vaccine making use of normal uridine. This vaccine is in many ways akin to other mRNA vaccines (from Pfizer–BioNTech and Moderna) and encodes a form of the coronavirus S protein that helps viral particles penetrate human cells [83].

The CureVac vaccine can be kept in a refrigerator longer than the mRNA vaccines manufactured by Pfizer–BioNTech and Moderna [84]. Depending on the environmental temperature, the stability of this vaccine is more flexible, with an expiry date of at least 3 months at a temperature of +5 °C (+41 °F), while the vaccine remains utilizable at room temperature for up to 24 h [85]. Likewise, this vaccine is administrated in two doses, with an interval of 29 days.

It is noteworthy that the efficacy of this novel mRNA vaccine has been reported to be far lower than that of the two other mRNA-based vaccines, offering only 47% protection against symptomatic COVID-19 infection. This low efficacy is presumed to be because of a number of factors such as dosage, immunogenicity, and SARS-CoV-2 variants [86].

#### ZyCoV-D

The ZyCoV-D vaccine, comprising circular strands of DNA known as plasmids, encodes the S protein and a promoter sequence of SARS-CoV-2 that turns the gene on. Upon entry of plasmids into the nuclei of cells, they are transformed into mRNA to travel into the cytoplasm, then this mRNA is translated into the S protein. The effect of this translation is to provoke a strong response to the protein by the immune system, thus producing tailored immune cells to fight future infections. Of note, despite the degradation of the plasmids over weeks to months, the immunity is long term [87].

It is noteworthy that ZyCoV-D is the first needle-free COVID-19 vaccine, while also being administered in three doses. It can be administrated via a needle-free injector, employing a small stream of fluid to enter the skin and deliver a shot to the correct region [88]. ZyCoV-D is deposited underneath the skin, where numerous immune cells devour vaccine particles [87].

To keep the ZyCoV-D vaccine utilizable, it can be stored at a temperature of 2–8 °C [88]; also, and most interestingly, it can be kept at 25 °C for at least 3 months [89]. A number of complaints have been reported by participants taking part in trial studies, namely injection-site pain, injection-site pruritus, pyrexia, arthralgia, and diarrhea. With regards to the efficacy of ZyCoV-D, the percentage protection of the vaccine has been found to be 67% in clinical trials [87].

#### Convidecia (Cansino)

The method used to make the Convidecia (Cansino) vaccine is to recombine the S-protein gene of the virus into the genetic material of a replication-deficient human adenovirus-5 (AdHu5). Upon injecting the vaccine, the recombinant adenovirus can carry the S-protein gene into the person’s cells, where the gene is used to make an S-protein antigen, thereby inducing an immune response in the vaccinated person [90]. It is of note that the adenovirus-based vector, of which AdHu5 is the epitome, has been deemed to be a safe and potent immunogenic vaccine delivery platform [91].

The Convidecia (Cansino) vaccine is recommended to be administered in one dose [90]. It is also important to mention that the efficacy of its immunity response against SARS-CoV-2 by T-cells commences from day 14 after vaccination, reaching its peak at 28 days after vaccination. The enzyme-linked immunosorbent assay (ELISA) technique was used to determine the number of S1 IgG antibodies produced in each participant. The results showed that the percentage of S1 IgG antibodies among the participants who received the Ad5-nCoV vaccine reached 11.11%. People with history of COVID-19 also had the highest levels of S1 IgG antibodies as a consequence of taking the Ad5-nCoV vaccine [92]. Note that one study showed that a single dose of Ad5-vectored vaccine can escalate elicitation of antibodies binding to RBD by fourfold among 94–100% of participants, and also increases living virus by fourfold among 50–75% of participants [93].

The first adverse reaction to vaccination was reported among 81.5% of participants [92], within 7 days after vaccination. Along with some symptoms such as fatigue and headache resulting from the vaccination, the most prevalent complaint was injection-site reaction and systematic reactions of pain and fever, respectively [93]. Incidentally, evidence derived from a phase II study yields to the prevailing notion that this vaccine exhibits an acceptable safety profile with no serious adverse events [91].

In addition to the findings described above, there are various concerns regarding this vaccine. For instance, since some people have immunity to Ad5, an immune response can be mounted against the vector, thus not delivering the S-protein gene into human cells [94]. After the vaccination, this vaccine declines the most powerful point of T-cell responses. This decline is because of preexisting adenovirus 5 neutralizing antibody levels. Preexisting antibodies can interfere with vaccine immunogenicity of adenovirus. This issue indicates that it is necessary to adopt either new vectors or heterogeneous prime-boost regimes for adenoviral vector-based vaccine production [95].

Another challenging factor is that advancing age limits the use and efficacy of this vaccine. It was found that participants aged under 55 showed remarkably lower immune responses against COVID-19 infection. Of note, in terms of adverse events after vaccination, grade 3 fever was more common among young compared with older persons [91].

#### Oxford–AstraZeneca

The Oxford–AstraZeneca vaccine is manufactured using a nonreplicating adenovirus vector platform, using a recombinant chimpanzee adenovirus (ChAdOx) [96, 97]. This method based on a nonreplicating adenovirus vector has remarkable benefits for protection against infectious diseases, in particular in terms of its safety profile, potential immunogenicity, capacity for high-titer growth, ease of manipulation, and compatibility with clinical manufacturing and thermostabilization procedures [95].

What is noticeable regarding the storage of the Oxford–AstraZeneca vaccine is that, unlike the other vaccines mentioned above, it can be stored in a regular refrigerator at temperatures of 2–8 °C. Some other protocols should also be followed to keep this vaccine safe: (1) the vaccine must not be frozen, (2) the vaccine’s vial should be discarded 6 h after first puncture, and (3) the vaccine can be stored at a temperature of 2–25 °C during use [80].

This vaccine is administrated in two doses, with an interval of 12 weeks or more. It was found that the percentage protection obtained from a single standard dose of vaccine can reach 76% against symptomatic COVID-19 over 90 days after vaccination. Accordingly, the efficacy can be escalated to  82.4% after administration of a second dose. Notably, the efficacy was shown to decline significantly, reaching 54.9%, if the interval between the two doses was shorter than 6 weeks. However, further study is required to determine how long protection from the Oxford–AstraZeneca vaccine may last [98]. Most interestingly, the efficacy was reported to be lower in the participates received two standard dose-Standard doses/Standard doses-(SD/SD cohort) than in the participants received a half dose as their first dose (low dose) and a standard dose as their second dose- Low doses/Standard doses-(LD/SD) one, depending on differences in both age and the interval between dosages [99]. Incidentally, it has been found whether test-positive cases have symptoms or not, the efficacy against any nucleic acid amplification among these patients can reach at 63.9% after a single standard dose, indicating to be possible diminishing viral transmission [100].

Statistically, persons given the vaccine were neither hospitalized nor died [101], and it was also found that a single dose of this vaccine could provide noticeable prevention against hospitalization for COVID-19, by 80% [102].

Regarding its safety and adverse effects, the Oxford–AstraZeneca vaccine has been vilified because of rare blood-clotting conditions that occurred in some participants, mostly women aged under 55 years [101], leading to the suspension of this vaccine by many countries [103]. According to some studies, it was pointed out that, albeit seldom reported, that the AstraZeneca vaccine ChAdOx1-S gives rise to cerebral venous thrombosis roughly 28 days after the first dose. Despite such reports, what ought to be considered is that the benefits of this vaccine outweigh the very low rate of thrombocytopenia/coagulation disorders or bleeding reported. This issue can also be demonstrated statistically in that the number of recipients who had thromboembolic disorders was negligible, reaching only 30 among 5 million participants [103]. Other side effects have been reported with the AstraZeneca vaccine, including fever and chills, weakness and fatigue, headache, nausea, muscle aches, and injection-site pain within 1 week of vaccination [104].

#### Janssen

The Janssen vaccine, also known as the Johnson and Johnson vaccine, is an adenovirus vaccine whose platform is a replication-incompetent human adenoviral type 26 vector [105]. A key part of the COVID-19 virus particle can be produced by modification of the DNA in the adenovirus, thus developing an immune response [81]. The Janssen vaccine is administered in one dose, and it can also be kept for approximately 2 years at a temperature of −20 °C (−4 °F) or stored for up to 3 months at 2–8 °C (36–46 °F) [106].

The booster dose of the Janssen vaccine may be given to persons aged over 18 years, at an interval of at least 2 months after the first dose. Of note, administration of this vaccine is not recommended for those who showed thrombocytopenia syndrome after their initial Janssen vaccine [107, 108]. To date, however, the incidence of thrombosis in either large arteries or veins after vaccination is trivial, reaching 3% according to a study carried out on 7.98 million recipients [105, 109]. The Janssen vaccine, although prohibited for a short period of time due to its side effects, has resulted in the prevention of numerous intensive care unit (ICU) admissions and deaths, in particular among people aged over 50 years, since its reintroduction [109].

Alongside adverse reactions at the injection site such as pain, redness of the skin, and swelling, the general side effects of the vaccine are fever, headache, weakness, and muscle aches. Furthermore, severe allergic reaction (defined as a reaction having symptoms such as hives, swelling, or wheezing) has been reported up to 1 h after a dose of the Janssen vaccine, which should be treated at once via either epinephrine or EpiPen [110]. There is a low probability that blood clots with low levels of platelet symptoms may commence roughly 1–2 weeks after administering this vaccine, being seen mostly among women aged 18–49 years. Moreover, Guillain–Barré syndrome, a neurological disorder in which the immune system harms neurons, has been reported approximately 42 days after administering the Janssen vaccine [106].

The efficacy of the Janssen COVID-19 vaccine reached 66.3% in clinical trials among participants lacking history of COVID-19 infection. The highest protection was seen 2 weeks after vaccination [108]. According to another study, this vaccine was able to protect recipients from moderate to severe symptoms of COVID-19 infection over 28 days after administering the vaccine, reaching 66%, and this efficacy was also found among those infected with emerging variants of the virus [111]. Incidentally, the immune system of immunocompromised persons receiving immunosuppressant therapy may show a diminished response to the Janssen COVID-19 vaccine [107].

#### Sputnik V (Gamaleya)

Sputnik V is another COVID-19 vaccine. Its platform is based on two adenoviruses: rAd26 and rAd5s [112]. Both components of the vaccine (adenovirus serotype 5 and 26) can be stored for up to 6 months at a temperature of −18 °C in the dark, whereas the lyophilized powder form of the vaccine can be kept in a regular refrigerator at a temperature of 2–8 °C, being available in a lyophilized (dry) form on international markets [113].

The Sputnik V vaccine is administered in two different doses, a first dose including serotype 26 and a second dose comprising serotype 5, with an interval of 21 days [113]. Upon injecting the vaccine, the gene from the adenovirus giving rise to COVID-19 is eradicated and replaced with a vector having a gene which produces the S protein of SARS-COV-2, leading to the production of antibodies by the immune system. Notably, the immune system can be augmented by injecting a second adenovirus vector 21 days afterwards, thus providing long-lasting immunity.

The Sputnik V vaccine has been shown to have high efficacy against COVID-19 infection, reaching 94%. This percentage efficacy was reported 21 days after the first dose of the vaccine, with 94% of naive recipients developing S-specific IgG antibodies. Interestingly, both the antibody levels and virus-neutralizing capacity were reported to be higher among previously infected persons receiving a single Sputnik V dose than among naive ones receiving the full, two-dose schedule [114].

Nonetheless, some flaws have been found in specific conditions; that is, the immunogenicity of this vaccine can be declined when faced with a high amount of Ad5-neutralizing antibody titers. The most common adverse reaction when administering this vaccine is flu-like illness [113]. There are finite data regarding the safety of adenovirus vector vaccines among pregnant women, although adverse events were not seen among pregnant mice that received adenovirus vector-based Zika vaccines [115, 116].

Regarding the Sputnik V vaccine, one should note that previous SARS-CoV-2-seropositive persons may need only one dose in terms of the level of IgG antibodies produced. Notably, the second dose was not able to increase the IgG response in the seropositive group [117].

#### Sinopharm (BBIBP)

The Sinopharm vaccine, also known as the BBIBP vaccine, is an inactivated SARS-CoV-2 virus candidate vaccine [118]. The virus is rendered hygienic using chemicals, namely formaldehyde, or heat, for use in this vaccine [119]. This vaccine makes use of Vero cells for growth. Thereafter, it is soaked in β-propionolactone to inactivate the virus by binding to its genes. The obtained inactivated viruses are then combined with the adjuvant aluminum hydroxide to improve the immunogenicity [90].

It is recommended that the Sinopharm vaccine be administrated in two doses with an interval of 3 weeks [90]. A prominent benefit of this vaccine is that it can be kept at normal fridge temperatures, in contrast to other vaccines that require extremely cold temperatures for storage [118].

According to studies, the efficacy of this vaccine has been reported to reach 78.1% against symptomatic SARS-CoV-2 infection after 112 days of receiving both doses. Also, it can be reach up to 78.7% against hospitalization [120]. In addition, a large multicountry phase III trial showed an increase in the efficacy of this vaccine by 79% within 14 or more days after taking the second dose [121].

The Sinopharm vaccine, like other vaccines, has been shown to cause some adverse reactions, the most common of which are fever, being mild and self-limiting. Dizziness, fatigue, headache, nausea, and allergic dermatitis are manifestations of such adverse reactions as reported by the World Health Organization (WHO) [122, 123].

#### Covaxin

Covaxin, code-named BBV152, is a COVID-19 vaccine developed using a platform based on whole-virion inactivated Vero cell technology [124, 125]. Covaxin mainly includes 6 μg of whole-virion inactivated SARS-CoV-2 antigen (strain NIV2020-770), and other, inactive components such as 250 μg aluminum hydroxide gel, 15 μg TLR 7/8 agonist (imidazoquinolinone), 2.5 mg TM2-phenoxyethanol, and phosphate buffer saline up to 0.5 ml [126].

Since the vaccine uses a complete infective SARS-CoV-2 viral particle comprising RNA surrounded by a protein shell, albeit modified, it cannot be replicated [124]. The formulation containing the TLR7/8 agonist also induced a distinct T helper cell 1 (Th1) biased antibody response with increased levels of SARS-CoV-2-specific interferon gamma (IFN-γ) + CD4 cells [127].

The Covaxine vaccine is administered in two doses with an interval of 28 days [124]. This vaccine also does not require subzero storage and reconstitution [128]. This vaccine can be kept at a temperature of 2–8 °C, as well as being shipped in a ready-to-use liquid formulation that facilitates its distribution. Notably, the Covaxine vaccine has a 28-day open vial policy, which is considered to be a unique characteristic, hence reducing vaccine wastage by roughly 10–30% [129].

It has been demonstrated that the efficacy of the Covaxine vaccine to prevent COVID-19 among persons without prior infection reaches 81% after injection of the second dose [129]. The most common adverse reaction was pain at the injection site, followed by headache, fever, and fatigue. Severe or life-threatening (grade 4 and 5) solicited adverse events were not reported [130, 131]. Incidentally, clinicians advise that breastfeeding and pregnant women should not receive this vaccine, nor persons with fever, bleeding disorders, blood thinner, or history of allergies [126].

#### CoviVac

The CoviVac vaccine is a “whole-virion” vaccine, made from a coronavirus that has been inactivated or stripped of its ability to replicate, including all the elements of the virus [132–134].

Each 0.5 ml dose consists of only 3 μg of the AYDAR-1 antigen of the SARS-CoV-2 strain, which is inactivated by β-propiolactone and excipients [135]. The CoviVac vaccine is administered in two doses with an interval of 14 days. It also can be kept at temperatures of 2–8 °C (36–46 °F) [136].

Aidar Ishmukhametov, the general director of the center that developed CoviVac, proclaimed that the efficacy of this vaccine is well above 80% against COVID-19 infection. This vaccine can also be effective against new variants such as alpha and delta [137]. Of note, this vaccine can be considered as a booster dose for persons initially receiving other vaccines [135].

In preclinical study, the short-term immunogenicity of the CoviVac vaccine has been evaluated in three animal models, immunized with different doses. As a result, NAbs were induced by the vaccine in all studied species. No significant difference emerged between the NAb titers induced by the different doses of the antigen over the first 2–4 weeks, while prominent differences started to appear from 5 weeks after immunization. This means that NAbs have a direct link to the protection against SARS-CoV-2. Therefore, this vaccine can produce a constant immune response in the form of both specific anti-SARS-CoV-2 IgG and NAbs in rodents and nonhuman primates. The NAb levels remain constant throughout 1 year [138].

#### Valneva

The Valneva vaccine, code-named VLA2001, is an inactivated Vero-cell-based vaccine against SARS-CoV-2. This vaccine includes an inactivated whole coronavirus particle with a high density of S protein to blend with alum, two adjuvants, and CpG 1018. The levels of antibody were shown to be increased via the adjuvant combination, far above those of alum-only formulations, in preclinical experiments [139]. The Valneva vaccine is recommended for administration in two doses with an interval of 21 days [140].

Regarding the efficacy of the Valneva vaccine, it has been proclaimed that this vaccine triggers a significantly stronger immune response, suggesting that the protection against COVID-19 in terms of the antibody response (having a neutralizing antibody seroconversion rate above 95%) would be better compared with the AstraZeneca vaccine. The study also showed that the Valneva vaccine resulted in significantly fewer adverse reactions, such as arm pain and fever [141].

#### Minhai

Minhai, trademarked as KCONVAC, is an inactivated COVID-19 vaccine which is inoculated in Vero cells for cultivation. The harvested virus can be inactivated via β-propiolactone, purified, and adsorbed onto aluminum hydroxide as adjuvant [142]. The Minhai vaccine is administered in two doses with an interval of 28 days [140]. In terms of adverse events (AEs) stemming from this vaccine, the most common injection-site and systemic AEs reported were pain and fatigue, respectively. One SAE (foot fracture) was reported in the 10-mg vaccine group. No participant discontinued the study because of an AE [143].

#### CoronaVac (Sinovac)

CoronaVac, also known as Sinovac, is an inactivated COVID-19 vaccine [140]. This vaccine is a Vero-cell-based, aluminum hydroxide-adjuvanted, β-propiolactone-inactivated vaccine based on the CZ02 strain [144].

Like many other vaccines, the dosage program for CoronaVac involves administration in two doses (0.5 ml) with an interval of 14 days [140], while its excipients are aluminum hydroxide, disodium hydrogen phosphate, sodium dihydrogen phosphate, sodium chloride, and water for injection; notably, it does not include preservatives. It has been noted that this vaccine can be kept at a temperature of 2–8 °C for up to 2 years [144].

According to studies carried out on the efficacy of this vaccine, CoronaVac shows high efficacy to protect recipients against symptomatic COVID-19 at 83.5% relative to placebo, and 100% for COVID-19-related hospitalization, at least 14 days after receiving the second dose among recipients aged 18–59 years. Furthermore, anti-RBD antibodies were promoted in 89.7% of participants, and 92.0% of those who were seropositive produced protective levels of neutralizing antibodies at least 14 days after receiving the second dose of the vaccine [145]. Further, in another study that included 10 million persons in Chile, the adjusted vaccine effectiveness was 65.9% for prevention of COVID-19, 87.5% for prevention of hospitalization, 90.3% for prevention of ICU admission, and 86.3% for prevention of COVID-19-related death [146].

The prevalent adverse reactions reported were injection-site pain and fever, of either mild (grade 1) or moderate (grade 2) severity. These reactions also occurred over 7 days after vaccination [147, 148]. Incidentally, an increased risk of Bell’s palsy has been reported as a result of CoronaVac vaccination [149].

#### EpiVacCorona

The EpiVacCorona vaccine includes synthesized immunogenic peptide corresponding to selected protective epitopes of the SARS-CoV-2 S protein, conjugated to the recombinant N protein, used as a carrier, and adjuvanted with aluminum hydroxide [150].

The short chemically synthesized fragments of the viral S protein peptides represent the protein regions containing B-cell epitopes that should be identified by the human immune system [150, 151]. The EpiVacCorona vaccine is administered in two doses with an interval of 21–28 days and also can be kept at a temperature of 2–8 °C [150].

According to the provisional results of the quality control review of the third phase of the vaccine’s clinical trials, the efficacy of the EpiVacCorona vaccine reached 79%. Notably, IgG coronavirus antibodies on the 42nd day after vaccination developed among 79% of volunteers compared with 11.6% of those who received placebo.

Adverse reactions of administering this vaccine include very mild and transient symptoms 1–2 days after vaccination. Local pain at the injection site after each injection was also reported as the most common adverse reaction of this vaccine [152].

#### Novavax

Novavax, also known as NVX-CoV2373, is a protein-based vaccine engineered from the genetic sequence of the first strain of SARS-CoV-2, the virus causing COVID-19 disease. NVX-CoV2373 is produced by a technology for generating antigen derived from the S protein, thus Novavax can develop immunogenic virus-like nanoparticles based on recombinant expression of the S-protein [153, 154]. This technology allows antigens encoded by the altered COVID-19 S protein gene (interfered with the vaccine) to attack cultured Sf9 insect cell lines (*Spodoptera frugiperda*). Upon infecting Sf9 insect cells, the antigens are expressed, creating recombinant nanoparticles containing S protein configurations [65].

In addition, NVX-CoV2373 can be stored in a standard refrigerator at a temperature of 2–8 °C. The efficacy of this vaccine against infection by the original SARS-CoV-2 variant reached 95.6% [154]. In another study on adults given two doses of this vaccine, the percentage protection was reported to be 89.7% [155].

Among the side effects of this vaccine, pain at the injection site was the most common complaint, in 29.3% and 51.2% after the first and second dose, respectively. Such an adverse local complication is reported to be more common among recipients aged 18–64 years than in individuals over 65 years [155].

#### ZIFIVAX

ZF2001, trade-named ZIFIVAX, is an adjuvant protein subunit COVID-19 vaccine that use a dimeric form of the RBD as the antigen, a harmless piece of the SARS-CoV-2 virus [156]. The recombined ZF2001 vaccine can encode the RBD antigen (residues 319–537, accession number YP_009724390) with two copies in tandem repeat dimeric form.

Interestingly, the ZF2001 vaccine is administered in three doses with an interval of 1 month between shots. One of the cited disadvantages of this vaccine is that this long interval may give rise to incomplete vaccination in some persons [65]. Regarding its efficacy, a phase III trial showed that this vaccine exhibits efficacy of 93% and 78% against the alpha and delta variants, respectively [157].

Most adverse events resulting from receiving this vaccine were mild or moderate. Injection-site pain, redness, and swelling were typical of such adverse events, predicted to be because of the alum-adjuvanted protein subunit vaccine. Also, these were transient and resolved over 3–4 days after vaccination [158].

#### Abdala

Abdala (technical name CIGB-66) is a protein subunit vaccine that is also based on the recombinant RBD subunit of the S protein, produced in *Pichia pastoris* yeast [159, 160].

It is administered in three doses with an injection schedule of 0–14–28 days. Based on reports, the Abdala vaccine achieves efficacy above 90% against severe illness and death [161].

It was found that both age and time affected the antibody titer level. For instance, after the second dose, lower antibody titers were shown. Furthermore, the rate of antibody titers derived from those aged under 50 years reduced rapidly with increasing age [162].

#### COVIran Barekat

COVIran Barekat is an inactivated virus-based vaccine that is also administered in two doses (a high dose of 5 μg and a low dose of 3 μg) with an interval of 28 days. This vaccine can be stored at standard refrigerator temperatures of 2–8 °C, which is considered to be one of its benefits [160]. As vaccines must be mixed with an adjuvant to achieve better absorption, this vaccine is mixed with 2% adjuvant^®^ Alhydrogel, too [163].

As a matter of interest, COVIran Barekat has shown high efficacy with respect to producing neutralizing antibodies; that is, production of neutralizing antibodies was reported among well above 93% of recipients in early trials [164]. The immunogenicity derived from this vaccine according to the conventional virus neutralizing test (cVNT) reached 93.5%. In terms of the geometric mean ratio of antibody titer in recipients, both a 76-fold rise in IgG anti-spike SARS-CoV-2 titer and a 36-fold growth in SARS-CoV-2 neutralizing antibodies were shown. Of note, hypotension, headache, and reduction of platelets were the typical adverse events reported, although mild adverse reactions were reported in phase I and II trials of the COVIran Barekat vaccine.

#### COVAX-19 (SpikoGen)

COVAX-19, also known as SpikoGen, is a protein subunit vaccine that is administered in two doses with an interval of 21 days. Its application in Australia will be based on interim data from a phase III SpikoGen trial that recruited 16,876 volunteers. Interim data showed that SpikoGen exceeded the 60% efficacy threshold as the primary endpoint of preventing symptomatic COVID-19 disease, based on a prespecified number of 88 PCR-confirmed infection events (https://www.clinicaltrialsarena.com/analysis/vaxine-australia-approval-covid-19-vaccine/).

The primary safety outcomes were the incidence of solicited adverse events up to 7 days after each dose and unsolicited adverse events up to 28 days after the each dose. Evaluation and comparison of individuals with seroconversion for IgG bAb against S protein and geometric mean titer (GMT) for IgG bonding antibody (bAb) against protein S were assessed on days 21 and 35 (https://www.irct.ir/trial/56287).

A comprehensive list of current COVID-19 vaccines is summarized in Table 2.

**Table 2:** Current COVID-19 vaccines

No.	Platform/vaccine type	Vaccine name	Manufacturer	Dosage	Schedule	Mode	Storage and temperature status	Targeted SARS-CoV-2 protein	Efficacy	Side effects	Current approvals	Refs.
1	RNA vaccine	BNT162b2 (Comimaty)	Pfizer/BioNTech + Fosun Pharma	Two doses (30 µg)	Day 0 + 21	IM	At temperature of −70 °C	Full-length S protein with proline substitutions	52.4% after one dose and 94.6% ≥ 7 days after two doses in adults	Fever, fatigue, headache, muscle pain, and chills were reported 1–2 days after vaccination	FDA, EUA, WHO, EUL, approved in 93 countries, CARPHA, EU recommendation, EMA approved	[165–168]
2	RNA vaccine	mRNA-1273	Moderna + National Institute of Allergy and Infectious Diseases (NIAID)	Two doses (100 µg)	Day 0 + 28	IM	Between −25 °C and −15 °C. Upon defrosting, kept at 2–8 °C for 30 min to 2 h, and also can be stored for up to 30 days prior to being punctured	Full-length S protein with proline substitutions	2.1% against symptomatic disease ≥ 14 days after one dose, 94.1% ≥ 14 days after two doses, and 100% against severe disease	Tiredness, headache, muscle pain, chills, fever, and nausea	FDA, EUA, WHO, EUL, approved in 57 countries , EMA approved	[85, 165, 167, 168]
3	RNA vaccine	CVnCoV (CureVac)	CureVac AG	Two doses (12 µg)	Day 0 + 28	IM	At least 3 months at temperature of +5 °C	LNP-encapsulated mRNA vaccine encoding the full-length, prefusion stabilized S protein	47% against symptomatic disease across all age groups and 15 variants, 53% against any disease severity, 77% against moderate and severe disease	Tiredness, headache, muscle pain, chills, fever	WHO, EUL (pending approval), not yet approved in any country	[85, 169]
4	RNA vaccine	ARCoV or ARCoVax	Academy of Military Science (AMS), Walvax Biotechnology, and Suzhou Abogen Biosciences	One dose (15 µg)	Day 0	IM	Not reported	LNP-encapsulated mRNA vaccine encoding the RBD of S protein	Not reported	Not reported	Not yet approved in any country	[170]
5	RNA vaccine	mRNA-1273.211	ModernaTX, Inc	One dose (50 µ)	Day 0	IM	Not reported	A multivalent booster candidate combining mRNA 1273 + mRNA 1273.351	Not reported	Not reported	Not yet approved in any country	[171]
6	RNA vaccine	mRNA-1273.351	Moderna + NIAID	One or two doses (20 or 50 µg)	Day 0, or day 0 + 28, or day 56 after second dose of mRNA1273	IM	At room temperature	Full-length prefusion stabilized S protein of SARS-CoV-2 B.1.351 variant	Not reported	Not reported	Not yet approved in any country	[171, 172]
7	RNA vaccine	ARCT-021	Arcturus Therapeutics	One or two doses ± booster dose (5 or 7.5 µg)	Day 0, or day 0 + 28, or day 0 + 28 ± 208 booster	IM	At room temperature	S protein	Not reported	Not reported	Not yet approved in any country	[173, 174]
8	RNA vaccine	MRT5500	Sanofi Pasteur + Translate Bio	Two doses (15, 45, or 135 µg)	Day 0 + 21	IM	At temperature of −20 °C	S protein	Not reported	Not reported	Not yet approved in any country	[175]
9	RNA vaccine	DS-5670a	Daiichi Sankyo Co., Ltd	Two doses (10, 30, 60, or 100 µg)	Day 0 + 21	IM	Not reported	Not reported	Not reported	Not reported	Not yet approved in any country	[173]
10	RNA vaccine	EXG-5003	Elixirgen Therapeutics, Inc	One dose	Day 0	ID	Temperature sensitive	Temperature-sensitive ssRNA vaccine expressing the RBD of S protein	Not reported	Not reported	Not yet approved in any country	[176]
11	RNA vaccine	LNP nCoVsaRNA (COVAC1)	Imperial College London	Two doses	ND	IM	Not reported	S protein	Not reported	Not reported	Not yet approved in any country	[177]
12	RNA vaccine	ChulaCov19 mRNA vaccine	Chulalongkorn University	Two doses (10, 25, 50, or 100 µg)	Day 0 + 21	IM	At 2–8 °C for 3 months and room temperature (25 °C) for 2 weeks	S protein	Not reported	Not reported	Not yet approved in any country	[178, 179]
13	RNA vaccine	PTX-COVID19-B	Providence Therapeutics	Two doses (16, 40, or 100 µg)	Day 0 + 28	IM	Not reported	Full-length membrane-anchored S protein	Not reported	Not reported	Not yet approved in any country	[180, 181]
14	RNA vaccine	CoV2 SAM (LNP)	GSK	Two doses (1.0 µg)	Day 0 + 30	IM	At temperature of 5 °C for at least 3 months	S protein	Not reported	Not reported	Not yet approved in any country	[182] NCT04758962
15	RNA vaccine	HDT-301	SENAI CIMATEC	Two doses (1, 5, or 25 µg)	Day 0 + 28	IM	At room temperature for at least 1 week	Full-length S protein	Not reported	Not reported	Not yet approved in any country	[183]
16	RNA vaccine	mRNA-1283	ModernaTX, Inc	One or two doses (10, 30, or 100 µg)	Day 0 or day 0 + 28	IM	At standard refrigerator temperature	RBD and NTD of S protein	Not reported	Not reported	Not yet approved in any country	[184, 185]
17	RNA vaccine	SW-0123	Shanghai East Hospital + Stemirna Therapeutics	Two doses	NR	IM	At temperature of under 4 °C	Not reported	Not reported	Not reported	Not yet approved in any country	[184, 186]
18	RNA vaccine	LNPnCOV saRNA-02 (COVAC Uganda)	MRC/UVRI and LSHTM Uganda Research Unit	Two doses (5.0 µg)	Day 0 + 28	IM	Not reported	S protein	Not reported	Not reported	Not yet approved in any country	NCT04934111
19	DNA vaccine	nCov vaccine (ZyCoVD)	Zydus Cadila	Three doses (1 or 2 mg)	Day 0 + 28 + 56	ID	At 2–8 °C, shown good stability at temperature of 25 °C for at least 3 months	S protein	In clinical trials in India, the efficacy is 66.6%	Fever, pain, feeling of illness	Not yet approved in any country	[187-189]
20	DNA vaccine	INO-4800 + electroporation	Inovio Pharmaceuticals + International Vaccine Institute + Advaccine Biopharmaceutica Co., Ltd	Two doses (1 mg)	Day 0 + 28	ID	At room temperature	S1 and S2 subunits of SARS-CoV-2 S protein	Not reported	Not reported	Not yet approved in any country	[190]
21	DNA vaccine	AG0301	AnGes + Takara Bio + Osaka University	Two doses (2 mg)	Day 0 + 14	IM	Not reported	S protein	Not reported	Not reported	Not yet approved in any country	[191]
22	DNA vaccine	GX-19	Genexine Consortium	Two doses	Day 0 + 28	IM	At room temperature	S protein	Not reported	Not reported	Not yet approved in any country	[192, 193]
23	DNA vaccine	Covigenix VAX 001	Entos Pharmaceuticals Inc	Two doses	Day 0 + 14	IM	At room temperature for a month and 4–8 °C for 1 year	Full-length S protein	Not reported	Not reported	Not yet approved in any country	NCT04591184
24	DNA vaccine	GLS-5310	GeneOne Life Science, Inc	Two doses (0.6 or 1.2 mg)	Day 0 + 56 or day 0 + 84	ID	At temperature of 2–8 °C	S protein and a second antigenic target of SARS-CoV-2	Not reported	Not reported	Not yet approved in any country	NCT04673149
25	DNA vaccine	COVIDeVax	Takis + Rottapharm Biotech	Two doses (0.5, 1, or 2 mg)	Day 0 + 28	IM	At room temperature	RBD of S protein	Not reported	Not reported	Not yet approved in any country	[194]
26	DNA vaccine	CORVax	Providence Health and Services	Two doses	Day 0 + 14	ID	Not reported	S protein +/− the combination of electroporated IL-12p70 plasmid	Not reported	Not reported	Not yet approved in any country	[195, 196]
27	DNA vaccine	bacTRL	Symvivo Corporation	One or two doses	Day 0 or day 0 + 28	Oral	At room temperature	S protein	Not reported	Not reported	Not yet approved in any country	[197, 198]
28	DNA vaccine	COVIGEN (COV-ALIA)	University of Sydney, Bionet Co., Ltd	Two doses (0.8, 2, or 4 mg)	Day 0 + 28	IM or ID	At room temperature	S protein	Not reported	Not reported	Not yet approved in any country	[199]
29	Viral vector (nonreplicating)	ChAdOx1 AZD1222	AstraZeneca + University of Oxford	Two doses (standard dose: 5 × 10^10^ viral particles, low dose: 2.2 × 10^10^ viral particles)	Day 0 + 28	IM	At room temperature up to 25 °C during use for 6 hours; at temperature of 2-8 ºC up to 48 hours during use	Chimpanzee adenovirus-vectored vaccine (ChAdOx1) expressing S protein	66.7–70.4% overall efficacy ≥ 14 days after two doses, 62.1% after two standard doses 76.0% after single low dose within 20–90 days, 90.0% after one low dose and one standard dose	Cerebral venous thrombosis roughly 28 days after taking the first dose was reported rarely. Chills, fatigue, headache, fever, nausea, muscle aches, malaise, and painful injection site	WHO, EUL, approved in 118 countries, endorsed by ART, CARPHA, EU recommendation, EMA approved	[200, 201]
30	Viral vector (nonreplicating)	Convidicea (Ad5nCoV)	CanSino Biological Inc. + Beijing Institute of Biotechnology	One dose (5 × 10^10^ viral particles per dose)	Day 0	IM	At temperature of 2–8 °C	Recombinant replication-defective human type 5 adenovirus (Ad5) expressing S protein	68.8% and 65.7% against symptomatic disease ≥ 14 days and ≥ 28 days after vaccination, respectively. 95.5% and 91.0% against severe disease ≥ 14 days and ≥ 28 days after vaccination, respectively	Fatigue, pain at vaccination site, muscle pain, and headache	WHO, EUL (approval pending), approved in eight countries	[202, 203]
31	Viral vector (nonreplicating)	Ad26.COV2.S	Janssen Pharmaceutical	One dose (5 × 10^10^ viral particles per dose)	Day 0	IM	At temperature of 2–8 °C	Recombinant replication-incompetent adenovirus serotype 26 (Ad26) vector encoding full-length and stabilized S protein	66.3–76.3% and 65.5–83.5% against moderate to severe/critical disease ≥ 14 days and ≥ 28 days after vaccination, respectively	Pain, redness, or swelling at injection site. Tiredness, headache, fever, muscle pain, or nausea may also occur	FDA, EUA, WHO, EUL, approved in 55 countries, endorsed by ART, EMA approved	[204–206]
32	Viral vector (nonreplicating)	GamCOVID- Vac (Sputnik V)	Gamaleya Research Institute + Health Ministry of the Russian Federation	Two doses (1 × 10^11^ viral particles per dose)	Day 0 + 21 (first: rAd26-S; second: rAd5-S)	IM	At normal fridge temperatures	Recombinant Ad26 and recombinant Ad5 encoding full-length S protein (rAd26-S and rAd5-S)	91.6% overall efficacy against symptomatic disease, 100% against moderate–severe disease, 73.1% after one dose, 91.1% after two doses	Flu-like illness, headache, fatigue, and injection-site reactions	WHO, EUL (approval pending), approved in 69 countries	[207–209]
33	Viral vector (nonreplicating)	GRAdCOV2	ReiThera + Leukocare + Univercells	One or two doses (1 × 10^11^ viral particles per dose)	Day 0 + 21	IM	Not reported	Replication-defective simian adenovirus (GRAd) encoding S protein	Not reported	Not reported	Not yet approved in any country	[210]
34	Viral vector (nonreplicating)	LV-SMENP-DC	Shenzhen Geno Immune Medical Institute	One dose (5 × 10^6^ cells of LV-DC vaccine and 1 × 10^8^ antigens specific CTLs)	Day 0	SC, IV	At temperature of 2–8 °C	Modified dendritic cells (DC) with lentivirus vectors (LV) expressing minigenes SMENP and immune modulatory genes. Cytotoxic T-cells (CTLs) are activated by LV-DC, presenting specific viral antigens	Not reported	Not reported	Not yet approved in any country	[211–213]
35	Viral vector (nonreplicating)	hAd5-SFusion + N-ETSD vaccine	ImmunityBio, Inc. + NantKwest, Inc	One dose (5 × 10^10^ IU/dose SC, 1 × 10^10^ IU/dose SL)	Day 0	SC, Oral, SL	At room temperature	Human second-generation adenovirus 5 (hAd5) encoding S and N antigens	Not reported	Not reported	Not yet approved in any country	[214–216]
36	Viral vector (nonreplicating)	AdCLDCoV19	Cellid Co., Ltd	One dose (2.5 × 10^10^, 5 × 10^10^, or 1 × 10^11^ virus particles per dose)	Day 0	IM	Not reported	Replication-defective human adenovirus type 5/35 vector expressing S protein	Not reported	Not reported	Not yet approved in any country	[195, 217]
37	Viral vector (nonreplicating)	COVIVAC	Institute of Vaccines and Medical Biologicals, Vietnam	Two doses (1 × 10^7^ IU, 5 × 10^7^ IU, or 1 × 10^8^ IU per dose)	Day 0 + 28	IM	At temperature of 2–8 °C	NDV expressing membrane-anchored prefusion-stabilized trimeric S protein +/− CpG 1018 adjuvant	Well above 80% against COVID-19 infection	Not reported	Not yet approved in any country	[112, 138]
38	Viral vector (nonreplicating)	MVA-SARS-2ST	Universitätsklinikum Hamburg-Eppendorf + German Center for Infection Research	Two doses (1 × 10^7^ IU, or 1 × 10^8^ IU per dose)	Day 0 + 28	IM	Not reported	MVA vector expressing stabilized S protein	Not reported	Not reported	Not yet approved in any country	[218]
39	Viral vector (nonreplicating)	MVA-SARS-2-S	University of Munich (Ludwig-Maximilians)	Two doses (1 × 10^7^ IU, or 1 × 10^8^ IU per dose)	Day 0 + 28	IM	Not reported	MVA vector expressing S protein	Not reported	Not reported	Not yet approved in any country	[219, 220]
40	Viral vector (nonreplicating)	VXA-CoV2-1	Vaxart	One or two doses (1 × 10^10^ IU, or 1 × 10^11^ IU per dose)	Day 0 or Day 0 + 28	Oral	At room temperature	Nonreplicating adenovirus vector expressing viral antigens and dsRNA adjuvant	Not reported	Not reported	Not yet approved in any country	[195, 212]
41	Viral vector (nonreplicating)	AdCOVID	Altimmune, Inc	One or two doses	Day 0 + NR	IN	At room temperature for over several months	Adenovirus expressing the RBD of S protein	Not reported	Not reported	Not yet approved in any country	[221]
42	Viral vector (nonreplicating)	COH04S1 (MVA-SARS-2-S)	City of Hope Medical Center + National Cancer Institute	Two doses (1 × 10^7^, 1 × 10^8^, or 2.5 × 10^8^ PFU per dose)	Day 0 + 28	IM	Not reported	Synthetic MVA carrying small pieces of SARS-CoV-2 DNA (the chemical form of genes)	Not reported	Not reported	Not yet approved in any country	[222]
43	Viral vector (nonreplicating)	ChAdV68- S ChAdV68S-TCE	Gritstone Oncology	Two or three doses (5 × 10^10^ or 1 × 10^11^ viral particles of ChAdV68 S, 10 µg or 30 µg SEM)	Day 0 + 28, or day 0 + 56, or day 0 + 112, or day 0 + 56 + 112	IM	Not reported	Chimpanzee adenovirus serotype 68 (ChAd) and self-amplifying mRNA (SAM) vectors expressing either S protein alone, or S protein with additional T-cell epitopes (TCE)	Not reported	Not reported	Not yet approved in any country	NCT04776317
44	Viral vector (nonreplicating)	SC-Ad6-1	Tetherex Pharmaceuticals Corporation	One or two doses	Day 0 or day 0 + 21	IM	At room temperature	Adenovirus vector vaccine	Not reported	Not reported	Not yet approved in any country	[223]
45	Viral vector (nonreplicating)	BBV154	Bharat Biotech International Limited	One or two doses (1 × 10^10^ viral particles per dose)	Day 0 or day 0 + 28	IN	Not reported	S protein	Not reported	Not reported	Not yet approved in any country	[221]
46	Viral vector (replicating)	DelNS12019nCoVRBDOPT1	University of Hong Kong, Xiamen University + Beijing Wantai Biological Pharmacy	Two doses (1 × 10^7^ EID50 and 1 × 10^7.7^ EID50)	Day 0 + 28	IN	Not reported	Genetically engineered live attenuated influenza virus vector expressing the RBD of S protein	Not reported	Not reported	Not yet approved in any country	[224]
47	Viral vector (replicating)	rVSV SARSCo-2-S	Institute for Biological Research	Two doses (1 × 10^5^, 1 × 10^6^, 1 × 10^7^, or 1 × 10^8^ PFU/ml)	Day 0 + 28	IM	Not reported	cDNA vector encoding the sequence of the N, P, M, and L genes of the VSV genome, and SARS-CoV-2 S protein	Not reported	Not reported	Not yet approved in any country	[225]
48	Viral vector (replicating)	AV-COVID-19	Aivita Biomedical, Inc. + National Institute of Health Research and Development + Ministry of Health Republic Indonesia	One dose (0.1, 0.33, or 1.0 mg)	Day 0	IM	At room temperature	Autologous dendritic cells loaded with antigens from SARS-CoV-2 +/− GM-CSF	Not reported	Not reported	Not yet approved in any country	[225]
49	Viral vector (replicating)	aAPC Covid19	Shenzhen Geno-Immune Medical Institute	Three doses	Day 0 + 14 + 28	SC	Not reported	Lentivirus vector system expressing viral minigenes to the artificial antigen-presenting cells (aAPCs)	Not reported	Not reported	Not yet approved in any country	[226–228]
50	Live-attenuated virus	COVIVAC	Codagenix, In. + Serum Institute of India	One or two doses	Day 0 or day 0 + 28	IN	At temperature of 2–8 °C	Whole SARS-CoV-2 with all viral proteins	Not reported	Not reported	Not yet approved in any country	[132]
51	Live-attenuated virus	MV-014212	Meissa Vaccines, Inc	One dose	Day 0	IN	At room temperature	RSV expressing SARS-CoV-2 S protein	Not reported	Not reported	Not yet approved in any country	[229]
52	Protein subunit	NVX-CoV2373	Novavax	Two doses (5 µg)	Day 0 + 21	IM	At temperature of 2–8 °C	S protein with Matrix-M adjuvant	89.7% against symptomatic disease ≥ 7 days after two doses. 100% against mild and severe disease	Injection-site pain and tenderness, as well as fatigue, headache, and muscle pain	WHO, EUL (approval pending), not yet approved in any country	[230, 231]
53	Protein subunit	ZF2001	Anhui Zhifei Longcom Biopharmaceutical + Institute of Microbiology, Chinese Academy of Sciences	Three doses (25 µg)	Day 0 + 30 + 93	IM	At temperature of 2–8 °C	RBD-dimer with alum adjuvant	Not reported	Not reported	China (EUA), Uzbekistan	[232, 233]
54	Protein subunit	VAT00008	Sanofi Pasteur + GSK	Two doses	Day 0 + 21	IM	Not reported	Monovalent and bivalent S protein with adjuvant	Not reported	Not reported	Not yet approved in any country	NCT04904549
55	Protein subunit	FINLAY-FR-2	Instituto Finlay de Vacunas	Two doses (25 µg)	Day 0 + 28, day 56 (booster dose)	IM	At temperature of 2–8 °C	Chemically conjugated RBD to tetanus toxoid plus adjuvant FINLAY FR-1A: dimeric RBD + alum adjuvant	62%	Injection-site pain and tenderness, fatigue, and fever	Not yet approved in any country	[140, 234]
56	Protein subunit	Recombinant SARSCoV-2 vaccine (Sf9 Cell)	West China Hospital + Sichuan University	Three doses	Day 0 + 28 + 42	IM	Not reported	RBD with alum adjuvant	Not reported	Not reported	Not yet approved in any country	[235]
57	Protein subunit	EpiVacCorona	Federal Budgetary Research Institution State Research Center of Virology and Biotechnology	Two doses	Day 0 + 21	IM	At temperature of 2–8 °C	Peptide antigens of SARS-CoV-2 proteins with alum adjuvant	The third phase of the vaccine’s clinical trials reported 79%	Local pain at injection site after each injection	Russia, Turkmenistan	[150, 236]
58	Protein subunit	CIGB-66	Center for Genetic Engineering and Biotechnology (CIGB)	Three doses	Day 0 + 14 + 28 or day 0 + 28 + 56	IM	At room temperature	RBD with aluminum hydroxide adjuvant	Well above 90% efficacy against severity and death	Not reported	Not yet approved in any country	[161, 237]
59	Protein subunit	NanoCovax	Nanogen Pharmaceutical Biotechnology	Two doses	Day 0 + 28	IM	At room temperature	Recombinant S protein with alum adjuvant	Nor reported	Not reported	Not yet approved in any country	[238]
60	Protein subunit	SCB-2019	Clover Biopharmaceuticals Inc. + GSK + Dynavax	Two doses	Day 0 + 21	IM	Stable at temperature of 2–8 °C for at least 6 months, and at room temperature and 40 °C for at least 1 month	Trimeric S protein with CpG 1018 and alum adjuvants	Not reported	Not reported	Not yet approved in any country	[239, 240]
61	Protein subunit	UB-612	Vaxxinity, Inc. + Diagnósticos da América S/A(DASA)	Two doses	Day 0 + 28	IM	At temperature of 2–8 °C	RBD of S protein	Not reported	Not reported	Not yet approved in any country	[241, 242]
62	Protein subunit	FINLAYFR-1	Instituto Finlay de Vacunas	Two doses	Day 0 + 28	IM	Not reported	RBD with adjuvant	Not reported	Not reported	Not yet approved in any country	[243]
63	Protein subunit	COVAX-19	Vaxine Pty Ltd. + CinnaGen Co	Two doses	Day 0 + 21	IM	Not reported	Recombinant S protein with Advax-CpG adjuvant	Not reported	Not reported	Not yet approved in any country	[244]
64	Protein subunit	MVC-COV1901	Medigen Vaccine Biologics + Dynavax + NIAID	Two doses	Day 0 + 28	IM	At temperature of 2–8 °C	Recombinant S protein with CpG 1018 and alum adjuvants	Not reported	Not reported	Not yet approved in any country	[245, 246]
65	Protein subunit	Razi Cov Pars	Razi Vaccine and Serum Research Institute	Three doses	Day 0 + 21 (IM) + 51 (IN)	IM and IN	Not reported	Recombinant S protein	Not reported	Not reported	Not yet approved in any country	[247]
66	Protein subunit	V-01	Guangdong Provincial Center for Disease Control and Prevention/ Gaozhou Center for Disease Control and Prevention	Two doses (10 or 25 µg)	Day 0 + 21	IM	Not reported	Recombinant S protein	Not reported	Not reported	Not yet approved in any country	[248]
67	Protein subunit	CIGB-669	Center for Genetic Engineering and Biotechnology (CIGB)	Three doses (50 µg RBD + 40 µg AgnHB)	Day 0 + 14 + 28 or day 0 + 28 + 56	IN	At room temperature	Recombinant RBD with AgnHB	Not reported	Not reported	Not yet approved in any country	[247, 249]
68	Protein subunit	KBP-COVID-19	Kentucky Bioprocessing Inc	Two doses (15 µg in phase I, 45 µg in phase II)	Day 0 + 21	IM	At room temperature	RBD of S protein	Not reported	Not reported	Not yet approved in any country	[250]
69	Protein subunit	BECOV2	Biological E. Limited	Two doses	Day 0 + 28	IM	Not reported	Recombinant RBD	Not reported	Not reported	Not yet approved in any country	[199]
70	Protein subunit	S-268019	Shionogi	Two doses	Day 0 + 21	IM	Not reported	Recombinant S protein	Not reported	Not reported	Not yet approved in any country	[195, 251]
71	Protein subunit	AKS-452	University Medical Center Groningen + Akston Biosciences Inc	One or two doses (22.5, 45, or 90 µg)	NR	SC or IM	Stable at room temperatures for at least 6 months	RBD-Fc fusion protein	Not reported	Not reported	Not yet approved in any country	[252]
72	Protein subunit	COVAC-1 and COVAC-2	University of Saskatchewan	Two doses (25, 50, or 100 µg)	Day 0 + 28	IM	Not reported	S1 protein with SWE adjuvant	Not reported	Not reported	Not yet approved in any country	[195]
73	Protein subunit	GBP510	SK Bioscience Co., Ltd. And CEPI	Two doses (10 or 25 µg)	Day 0 + 28	IM	At temperature of 2–8 °C	Recombinant RBD with AS03 aluminum hydroxide adjuvant	Not reported	Not reported	Not yet approved in any country	[253]
74	Protein subunit	QazCoVacP	Research Institute for Biological Safety Problems	One or two doses	Day 0 + 21	IM	At temperature of 2–8 °C	Not reported	Not reported	Not reported	Not yet approved in any country	[160, 237]
75	Protein subunit	EuCorVac19	POP Biotechnologies and EuBiologics Co., Ltd	Two doses	Day 0 + 21	IM	Not reported	Recombinant S protein with an adjuvant	Not reported	Not reported	Not yet approved in any country	[199]
76	Protein subunit	Recombinant (CHO cell)	National Vaccine and Serum Institute, China	Three doses	Day 0 + 30 + 60	IM	Not reported	Recombinant SARS-CoV-2	Not reported	Not reported	Not yet approved in any country	[254] NCT04869592
77	Protein subunit	SARS-CoV-2 Sclamp vaccine	University of Queensland + Syneos Health + CEPI	Two doses (5, 15, or 45 µg)	Day 0 + 28	IM	At temperature of 2–8 °C	Recombinant S protein with MF59 adjuvant	Not reported	Not reported	Not yet approved in any country	[255, 256]
78	Protein subunit	IMP CoVac-1	University Hospital Tuebingen	One dose (500 µL)	Day 0	SC	At room temperature	SARS-CoV-2 HLA-DR peptides	Not reported	Not reported	Not yet approved in any country	[199, 257] NCT04546841
79	Protein subunit	AdimrSC-2f	Adimmune Corporation	NR	NR	NR	At room temperature	Recombinant RBD with alum adjuvant	Not reported	Not reported	Not yet approved in any country	[258, 259]
80	Protein subunit	NBP2001	SK Bioscience Co., Ltd	Two doses (30 or 50 µg)	Day 0 + 28	IM	At temperature of 2–8 °C	Recombinant RBD protein with alum adjuvant	Not reported	Not reported	Not yet approved in any country	[199]
81	Protein subunit	ReCOV	Jiangsu Rec-Biotechnology	Two doses (20 or 40 µg)	Day 0 + 21	IM	Not reported	Recombinant two component S and RBD protein	Not reported	Not reported	Not yet approved in any country	NCT05084989
82	Protein subunit	SpikeFerritin-Nanoparticle (SpFN)	Walter Reed Army Institute of Research (WRAIR)	Two or three doses (25 or 50 µg)	Day 0 + 28 + 180	IM	At room temperature	S proteins with a liposomal formulation QS21 (ALFQ) adjuvant	Not reported	Not reported	Not yet approved in any country	[260, 261]
83	Protein subunit	CoVepiT	OSE Immunotherapeutics	One or two doses	Day 0 or Day 0 + 21	SC	At room temperature	Target 11 viral protein (S, M, N, and several nonstructural proteins)	Not reported	Not reported	Not yet approved in any country	NCT04885361
84	Protein subunit	CoV2-OGEN1	VaxForm	One or two doses (50, 100, or 200 µg)	Day 0 or Day 0 + 14	Oral	At room temperature	Recombinant RBD protein	Not reported	Not reported	Not yet approved in any country	NCT04893512
85	Virus-like particle	CoVLP	Medicago Inc	Two doses (3.75 µg)	Day 0 + 21	IM	At temperature of 2–8 °C	Trimeric S protein with AS03 adjuvant	Not reported	Not reported	Not yet approved in any country	[262, 263]
86	Virus-like particle	RBD SARS-CoV-2 HBsAg VLP	Serum Institute of India + Accelagen Pty + SpyBiotech	Two doses (5 or 25 µg)	Day 0 + 28	IM	At temperature of 2–8 °C	RBD conjugated to hepatitis B surface antigen	Not reported	Not reported	Not yet approved in any country	[264, 265]
87	Virus-like particle	VBI-2902a	VBI Vaccines Inc	Two doses (5 or 10 µg)	Day 0 + 28	IM	At temperature of 2–8 °C, and at room temperature for 10 min	Enveloped S glycoprotein with aluminum phosphate adjuvant	Not reported	Not reported	Not yet approved in any country	[184, 266]
88	Virus-like particle	SARSCoV-2 VLP	Scientific and Technological Research Council of Turkey	Two doses	NR	SC	At temperature of 2–8 °C, and at room temperature for 1 h		Not reported	Not reported	Not yet approved in any country	[199, 267]
89	Virus-like particle	ABNCoV2	Radboud University	Two doses	Day 0 + 28	IM	At room temperature	Capsid virus-like particle (cVLP) +/− adjuvant MF59	Not reported	Not reported	Not yet approved in any country	[268–270]
90	Inactivated virus	CoronaVac	Sinovac Research and Development Co., Ltd	Two doses (3 µg)	Day 0 + 14	IM	At temperature of 2–8 °C	Whole inactivated SARS-CoV-2 with aluminum hydroxide adjuvant	83.5% against symptomatic disease ≥ 14 days after two doses	Injection-site pain and fever	Not yet approved in any country	[145, 271, 272]
91	Inactivated virus	BBIBPCorV	Sinopharm + China National Biotec Group Co + Beijing Institute of Biological Products	Two doses (4 µg)	Day 0 + 21	IM	At normal fridge temperatures	Whole inactivated SARS-CoV-2	78.1% against symptomatic disease ≥ 14 days after two doses, and 79% against hospitalization	Dizziness, fatigue, headache, nausea, vomiting, allergic dermatitis, and fever	WHO and 83 countries	[118, 273]
92	Inactivated virus	Inactivated SARSCoV-2 vaccine (Vero cell)	Sinopharm + China National Biotec Group Co + Wuhan Institute of Biological Products	Two or three doses (5 µg)	Day 0 + 21 + 42 or 111 or 171	IM	At temperature of 2–8 °C	Whole inactivated SARS-CoV-2 with aluminum hydroxide adjuvant	72.8% against symptomatic disease ≥ 14 days after two doses, and 79% against hospitalization	Injection-site pain and fever	WHO and 31 countries	[274, 275]
93	Inactivated virus	Inactivated SARSCoV-2 vaccine (Vero cell)	Institute of Medical Biology + Chinese Academy of Medical Sciences	Two doses (50, 100, or 150 EU)	Day 0 + 14	IM	At temperature of 2–8 °C	Whole inactivated SARS-CoV-2 with Al(OH)_3_ adjuvant	Not reported	Not reported	Not yet approved in any country	NCT05164731
94	Inactivated virus	QazCovidin	Research Institute for Biological Safety Problems, Rep of Kazakhstan	Two doses	Day 0 + 21	IM	At temperature of 2–8 °C	Whole inactivated SARS-CoV-2	Clinical trials in Kazakhstan showed efficacy of 96%	No serious or severe adverse events were recorded	Republic of Kazakhstan	[276, 277]
95	Inactivated virus	BBV152 (COVAXIN)	Bharat Biotech International Limited	Two doses (3 or 6 µg)	Day 0 + 14	IM	At temperature of 2–8 °C, unpunctuated vials can be stored at 9–25 °C for up to 12 h	Whole inactivated SARS-CoV-2 with Algel-IMDG adjuvant	77.8% against symptomatic disease, 93.4% against severe disease, 63.6% against asymptomatic disease	Cough, fever or chills, shortness of breath, tiredness, nasal congestion, headache, conjunctivitis, muscle or body pain, sore throat, loss of taste or smell, diarrhea, and nausea or vomiting	WHO EUL (approval pending), approved in nine countries	[278–281]
96	Inactivated virus	Inactivated SARSCoV-2 (Vero cell)	Shenzhen Kangtai Biological Products Co., Ltd	Two doses	Day 0 + 28	IM	At temperature of 2–8 °C	Whole inactivated SARS-CoV-2	Not reported	Not reported	China	[140] NCT04852705
97	Inactivated virus	VLA2001	Valneva, National Institute for Health Research, United Kingdom	Two doses	Day 0 + 21	IM	At temperature of 2–8 °C	Whole inactivated SARS-CoV-2 with high S protein density, in combination with two adjuvants, alum and CpG 1018	Not reported	Not reported	Not yet approved in any country	[282, 283]
98	Inactivated virus	ERUCOV-VAC (TURKO-VAC)	Erciyes University + Health Institutes of Turkey	Two doses (3 µg)	Day 0 + 28	IM	At temperature of 2–8 °C and at room temperature for up to 24 h	Whole inactivated SARS-CoV-2	Not reported	Not reported	Not yet approved in any country	[195, 284]
99	Inactivated virus	COVIran Barekat	Shifa Pharmed Industrial Co	Two doses (5 µg)	Day 0 + 28	IM	At temperature of 2–8 °C	Whole inactivated SARS-CoV-2	Well above 93% has been reported	Hypotension, headache, and diminution of platelets were typical adverse events reported	Iran	[163, 164, 277, 285, 286]
100	Inactivated virus	FAKHRAVAC (MIVAC)	Organization of Defensive Innovation and Research	Two doses (10 µg)	Day 0 + 14	IM	Not reported	Whole inactivated SARS-CoV-2	Not reported	Not reported	Not yet approved in any country	[287, 288]
101	Inactivated virus	Inactivated (NDV-based) chimeric vaccine	Government Pharmaceutical Organization (GPO) + PATH + Dynavax	Two doses	Day 0 + 28	IM	At room temperature	Whole inactivated NDV chimera stably expressing membrane anchored SARS-CoV-2 S protein +/− CpG 1018 adjuvant	Not reported	Not reported	Not yet approved in any country	[199, 289]
102	Inactivated virus	KD-414	KM Biologics Co., Ltd	Two doses	Day 0 + 28	IM	Not reported	Whole inactivated SARS-CoV-2	Not reported	Not reported	Not yet approved in any country	[290] jRCT2071210081
103	Inactivated virus	Koçak-19	Kocak Farma, Turkey	Two doses (4 or 6 µg)	Day 0 + 21	IM	At room temperature	Whole inactivated SARS-CoV-2 with adjuvant	Not reported	Not reported	Not yet approved in any country	NCT04838080
104	Inactivated virus	Adjuvanted inactivated vaccine	Scientific and Technological Research Council of Turkey (TÜBITAK)	Two doses (10 µg–3 M or 20 µg–6 M)	Day 0 + 20	SC	Not reported	Whole inactivated SARS-CoV-2 with CpG ODN adjuvant	Not reported	Not reported	Not yet approved in any country	[291]
105	Inactivated virus	Live recombinant (rNDV) vector vaccine	Laboratorio Avi-Mex	Two doses	Day 0 + 21	IM or IN	Not reported	Live recombinant NDV vector expressing SARS-CoV-2 S protein	Not reported	Not reported	Not yet approved in any country	NCT04871737

### Reported SARS-CoV-2 variants

To date, SARS-CoV-2 variants have been categorized into two types, viz. variants of concern (VOCs) and variants of interest (VOIs). Alpha (B.1.1.7), beta (B.1.351), gamma (P.1), delta (B.1.617.2), and omicron (B.1.1.529) are VOCs, while the VOIs include both lambda (C.37) and mu (B.1.621). Such mutation is thought to enable the virus to escape some of the immune response [292]. Some important feature of SARS-CoV-2 variants are discussed in the paragraphs below.

#### Alpha (B.1.1.7)

Alpha (B.1.1.7), also known as VOC 202012/01 or 20B/501Y.V1, was discovered on 14 December 2020. It can spread 56% faster than other lineages, and also the alpha variant viral load in nasopharyngeal swabs has been reported to be higher than that of the wild-type strain. Accordingly, the rate of death associated with this variant has been estimated at 35%, compared with the original strain, whereby this variant can give rise to severe disease [293]. The mutation in the S protein of the alpha variant is sevenfold (N501Y, A570D, D614G, P681H, T716I, S982A, and D1118H) plus two deletions (H69-V70del and Y144del) [293].

#### Beta (B.1.351)

Beta (B.1.351) is another variant, identified on 18 December 2020. Even though higher transmission rates were reported as a notable feature of the beta variant, evidence of greater virulence or disease severity has not been reported to date [293]. The mutation of the S protein in the beta variant is ninefold (L18F, D80A, D215G, R246I, K417N, E484K, N501Y, D614G, and A701V) plus one deletion (LAL 242–244 del) [293].

Of note, it has been pointed out that the efficacy of some COVID-19 vaccines such as those from Novartis, Johnson & Johnson, and AstraZeneca–Oxford was remarkably diminished in South Africa where this variant was prevalent, raising concerns about the protection offered by vaccines on the market and clinical trials against the beta variant [294].

#### Gamma (p.1)

Gamma (p.1) was detected on 6 January 2021. There is a strong likelihood that the gamma variant is more resistant than the beta variant to neutralization via both monoclonal antibodies and vaccine sera. Some preprint studies, nonetheless, have shown that the gamma variant is less resistant to antibody responses stemming from either former illness or vaccination compared with the beta variant [295]. The mutation in the S protein of the gamma variant is 12-fold (L18F, T20N, P26S, D138Y, R190S, K417T, E484K, N501Y, D614G, H655Y, T1027I, and V1176F) [293].

#### Delta (B.1.617.2)

The delta (B.1.617.2) variant became the main transmitted SARS-CoV-2 variant from 28 June to 11 July 2021 according to complete SARS-CoV-2 genome sequencing during the period, accounting for 68.3% of isolates. The mutation of the S protein of the delta variant is fourfold (L452R, T478K, D614G, and P681R) [296].

#### Omicron (B.1.1.529)

Omicron (B.1.1.529) is the newest variant as of December 2021. Its level of variation has led to concerns regarding its transmissibility, immune system evasion, and vaccine resistance, although initial studies have shown that this variant gives rise to less serious disease than previous strains. Omicron is thought to be far more contagious, spreading much quickly compared with other variants. Furthermore, the omicron variant has the ability to spread around 70 times faster than any former variants in the bronchi, notwithstanding its ability to penetrate into deep lung tissue; hence, there is a noticeable decline in the risk of severe disease requiring hospitalization for most people. This variant has a total of 60 mutations compared with the original Wuhan variant [158, 297].

### Impact of variants on vaccine efficacy

Various strategies are being employed by the six COVID-19 vaccines currently in use around the world. Making use of prefusion “locked” S plays a pivotal role in producing the levels of neutralizing antibodies; that is, vaccines not utilizing prefusion “locked” S are expected to produce lower levels of neutralizing antibodies, hence having lower efficacy against COVID-19 infection [298].

#### Pfizer–BioNTech

It has been found that the Pfizer–BioNTech vaccine has the ability to induce neutralization immune sera against the alpha (B.1.1.7) variant. Of note, this ability against the beta (B.1.351) variant was far lower in comparison with the alpha (B.1.1.7) variant. According to a study carried out by Gavin et al. based on live virus, a 7.6-fold reduction was seen in the neutralization titers against the beta (B.1.351) variant compared with 3.3 against the alpha (B.1.1.7) strain [294]. A study carried out in Qatar showed that, despite the reduction in vaccine effectiveness observed after one dose of the Pfizer vaccine against the beta variant, receipt of a second dose increased the protection by 75% against beta-induced infection and 97.4% against severe illness [299].

In one study conducted by the University of Texas and the production company Pfizer–BioNTech, three SARS-CoV-2 virus mutants were engineered, namely N501Y, Δ69/70 + N501Y + D614G, and E484K + N501Y + D614G. The implication of this study was that the neutralization titers of 20 human sera administered Pfizer vaccine were at 0.81–1.46 fold, compared with the wild-type strain titers. Thus, this vaccine has low efficacy  to protect recipients against viruses bearing these mutations [300]. The efficacy of the Pfizer vaccine in the UK after one shot against the alpha and delta variants reached 51.1% and 33.5%, respectively.

Recent studies have shown that the efficacy of the Pfizer vaccine against the delta variant is 30% and 88% after one and two doses, respectively. Furthermore, this is enhanced to protect recipients against alpha and delta after administering the second dose, reaching 93.4% and 87.9%, consecutively [296].

Regarding the impact on hospitalization of the Pfizer vaccine, the percentage protection from this vaccine against the alpha and delta variants was reported to be similar after administering the first and second dose respectively, being 94% and 96%, [295].

It is worth noting that this vaccine, due to the recognition of 80% of the epitopes in the S proteins by CD8^+^ T cells, may not be effective against mutations in the omicron variant.

Further, data indicate that a third dose of the Pfizer–BioNTech vaccine increases the neutralizing antibody titers by 25-fold compared with two doses against the omicron variant, even though two doses may still offer protection against severe disease caused by the omicron strain [301].

#### Moderna

It has been found that the efficacy of two doses of the Moderna vaccine against the alpha and delta variants can reach 98.4% and 86.7%, respectively. Further, the efficacy of this vaccine to protect persons administered two doses of the vaccine against hospitalization reached 97.5%, while no individual was reported to be hospitalized with other variants [302]. Among persons receiving a second dose of the Moderna vaccine, the effectiveness against omicron was 36.7%, compared with the unvaccinated [303].

#### Oxford–AstraZeneca

The neutralization titer sera against the alpha (B.1.1.7) strain obtained from the second dose of the Oxford–AstraZeneca vaccine at 14 and 28 days were shown to decline by 2.5- and 2.1-fold, respectively. This rate was also reported to decrease by ninefold for the beta (B.1.351) variant. These results indicate that the protection offered by the Oxford–AstraZeneca vaccine against the beta (B.1.351) variant is far lower than against the Alpha (B.1.1.7) variant [294]. In another study also, plasma taken before the first dose of the Oxford–AstraZeneca vaccine showed minimal or no neutralization of B.1.351 viruses [304]. Accordingly, no protective effect was seen against the omicron variant from 15 weeks after the second dose among those who had received two doses of the Oxford–AstraZeneca vaccine. Interestingly, the efficacy increased to reach 71.4% among participants who received the Oxford–AstraZeneca vaccine as the primary course, at 2 weeks after receiving a Pfizer–BioNTech booster dose [305].

#### Sputnik V

According to a study to determine the efficacy of the Sputnik V vaccine, it was pointed out that the neutralizing antibodies declined by 7.13-fold against the omicron variant compared with other reported variants [306]. Also, changes in the level of serum neutralizing antibodies of those vaccinated with Sputnik V against alpha, beta, gamma, and delta were shown to be remarkable for the beta variant [307].

#### Novavax

The Novavax vaccine has been shown to exhibit high efficacy against the B.1.1.7 variant, reaching 85.6%. Its efficacy, however, was reported to be much lower against the B.1.351 variant, viz. 49.4% [308, 309]. In another study, the preliminary efficacy of the Novavax vaccine against the beta lineage was reported to be 51% [310]. Although the Novavax vaccine manufacturer has proclaimed that this vaccine provides high protection against the omicron variant, further studies are imperative to prove this claim.

#### Sinopharm

Data have revealed that the neutralizing activity diminished swiftly by 8–9 months after two doses of vaccination, whereby a third booster dose is indispensable to extend the duration of the humoral response against emerging variants. The neutralization sensitivity has been shown to decline as a result of exposure to the omicron variant compared with the wild-type strain of the booster elicited serum, with a reduction of about 20.1-fold [311].

## The future of COVID-19 vaccines

According to a panel of experts who advise the FDA, the next generation of COVID-19 vaccines not only should be able to fight off a new strain but also may presumably be administered annually. However, it should be considered that the evolution of the virus will dictate how we respond in terms of additional vaccine doses. This means that the virus itself will dictate vaccination plans. Of note, the efficacy of vaccination, along with therapeutic agents such as monoclonal antibodies, that remarkably diminish both hospitalization and death is promising. Also, developments in vaccine manufacture may bring the burden of COVID-19 infection to levels that are equivalent to or even lower than influenza [312, 313]. However, further studies are required to realize this optimist viewpoint regarding the future of COVID-19 and the efficacy of COVID-19 vaccines.

The efficacy of vaccines is measured by using antibody levels or titers as surrogate biomarkers. One concern is that antibody levels derived from COVID-19 vaccines dwindle over time, albeit remaining detectable at up to 8 months in the majority of recovered patients [314]. Although there is insufficient evidence that this decline in antibody levels is linked to a drop in COVID-19 protection, this decline is considered to be a matter of concern, prompting consideration of additional vaccine booster doses [315]. Such booster vaccination may augment neutralizing antibody titers, in particular among immunocompromised individuals, for whom this is vital because of an inadequate immune response following two doses.

However, booster vaccination displays similar vaccine-related side effects as seen with the first and second dose [316]. By comparison with the second dose, local reactions such as pain or swelling were slightly more common while systemic reactions such as fever or headache were less common after a third dose of the Pfizer–BioNTech vaccine. Statistically, injection-site reactions were reported by 79.4% after a third vaccine dose, compared with 77.6% after a second dose, while systemic reactions were reported by 74.1% after the third dose, compared with 76.5% after the second [317]. Another issue is that vaccines are not ubiquitous, in particular in developing countries. This is why next-generation vaccines that maintain long-lasting high-titer neutralizing antibodies, as well as having reduced adverse effects, must be produced.

Recently, a next-generation SARS-CoV-2 protein vaccine, an interferon (IFN)-armed receptor binding domain (RBD)-dimer fusion protein vaccine (I-P-R-F, or V-01 for short), was introduced. V-01 generated high levels of neutralizing antibody titers with low toxicity, giving rise to complete antiviral protection and even viral clearance from the upper respiratory tract 24 h after infection in vaccinated monkeys [318]. In clinical trials, participants who received V-01 presented three- to fourfold higher serum neutralizing antibody titers to the original SARS-CoV-2 strain than convalescent sera. V-01 has also been shown to have an excellent safety profile in both young and old groups in phase I and II trials, respectively [319, 320]. This next-generation protein vaccine V-01 can thus be considered to be a potent candidate to counter future COVID-19 variants and may also be an effective alternative to booster dose vaccines to extend neutralizing antibody titers [321]. These findings must be accompanied by further studies to reach a consensus on this issue.

## Conclusions

The latest findings regarding both drugs and vaccines to treat and prevent COVID 19 infection are described herein. More than 100 vaccines, classified into various platforms based on RNA vaccines, adenovirus vector vaccines, subunit (protein-based) vaccines, and inactivated virus vaccines, are under study, among which only a few have been approved by the WHO for prevention and treatment of COVID-19. Since variants in the SARS-CoV-2 genome can change the pathogenic potential of virus and hinder both drug and vaccine development, ongoing continuous surveillance is required to monitor long-term immunity and safety concerns, even though the results of clinical trials of current vaccines as well as some repurposed drugs have shown promising results among COVID-19 patients. Overall, the area of therapeutics, diagnostics, and vaccines for COVID-19 infection is continuing to develop, and we hope that promising results are on the horizon.

## Data Availability

The datasets used and/or analyzed during the current study are available from the corresponding author on reasonable request.

## Abbreviations

ACE2: Angiotensin-converting enzyme 2
APCs: Antigen-presenting cells
BCR: B-cell receptor
cVNT: Conventional virus neutralizing test
DCs: Dendritic cells
DPP4: Dipeptidyl peptidase-4
ERGIC: Endoplasmic reticulum–Golgi intermediate compartment
HLA: Human leukocyte antigen
IFNγ: Interferon gamma
ITP: Immune thrombocytopenia
MHC II: Major histocompatibility complex class II
Nk: Natural killer
Nabs: Neutralizing antibodies
NF-κB: Nuclear factor kappa-light-chain-enhancer of activated B cells
nCoV-2019: Novel coronavirus 2019
RBD: Receptor binding domain
Tregs: Regulatory T-cells
PRRs: Pattern recognition receptors
RdRp: RNA-dependent RNA polymerase
TCR: T-cell receptor
Th1: T helper 1
TLR: Toll-like receptor
TGF-β: Transforming growth factor beta
TNF: Tumor necrosis factor
VOCs: Variants of concerns
VOIs: Variants of interests
